# RNA Nanotherapeutics for the Amelioration of Astroglial Reactivity

**DOI:** 10.1016/j.omtn.2017.11.008

**Published:** 2017-11-24

**Authors:** Jayden A. Smith, Alice Braga, Jeroen Verheyen, Silvia Basilico, Sara Bandiera, Clara Alfaro-Cervello, Luca Peruzzotti-Jametti, Dan Shu, Farzin Haque, Peixuan Guo, Stefano Pluchino

**Affiliations:** 1 Department of Clinical Neurosciences, Division of Stem Cell Neurobiology, Wellcome Trust-Medical Research Council Stem Cell Institute and NIHR Biomedical Research Centre, University of Cambridge, Cambridge, UK; 2 Department of Diagnostics and Public Health, University of Verona, Verona 37134, Italy; 3 Department of Life Sciences, University of Trieste, Trieste 34127, Italy; 4 College of Pharmacy, Division of Pharmaceutics and Pharmaceutical Chemistry, The Ohio State University, Columbus, OH, USA; 5 College of Medicine, Department of Physiology and Cell Biology, The Ohio State University, Columbus, OH, USA; 6 Dorothy M. Davis Heart and Lung Research Institute, The Ohio State University, Columbus, OH, USA; 7 NCI Comprehensive Cancer Center, The Ohio State University, Columbus, OH, USA; 8 Center for RNA Nanobiotechnology and Nanomedicine, The Ohio State University, Columbus, OH, USA

**Keywords:** RNA nanotherapeutics, astrocytes, Lipocalin2, astrogliosis, siRNA, pRNA, neuroinflammation, spinal cord injury

## Abstract

In response to injuries to the CNS, astrocytes enter a reactive state known as astrogliosis, which is believed to be deleterious in some contexts. Activated astrocytes overexpress intermediate filaments including glial fibrillary acidic protein (GFAP) and vimentin (Vim), resulting in entangled cells that inhibit neurite growth and functional recovery. Reactive astrocytes also secrete inflammatory molecules such as Lipocalin 2 (Lcn2), which perpetuate reactivity and adversely affect other cells of the CNS. Herein, we report proof-of-concept use of the packaging RNA (pRNA)-derived three-way junction (3WJ) motif as a platform for the delivery of siRNAs to downregulate such reactivity-associated genes. *In vitro*, siRNA-3WJs induced a significant knockdown of *Gfap*, *Vim*, and *Lcn2* in a model of astroglial activation, with a concomitant reduction in protein expression. Knockdown of *Lcn2* also led to reduced protein secretion from reactive astroglial cells, significantly impeding the perpetuation of inflammation in otherwise quiescent astrocytes. Intralesional injection of anti-*Lcn2*-3WJs in mice with contusion spinal cord injury led to knockdown of Lcn2 at mRNA and protein levels *in vivo*. Our results provide evidence for siRNA*-*3WJs as a promising platform for ameliorating astroglial reactivity, with significant potential for further functionalization and adaptation for therapeutic applications in the CNS.

## Graphical Abstract

**Figure:**
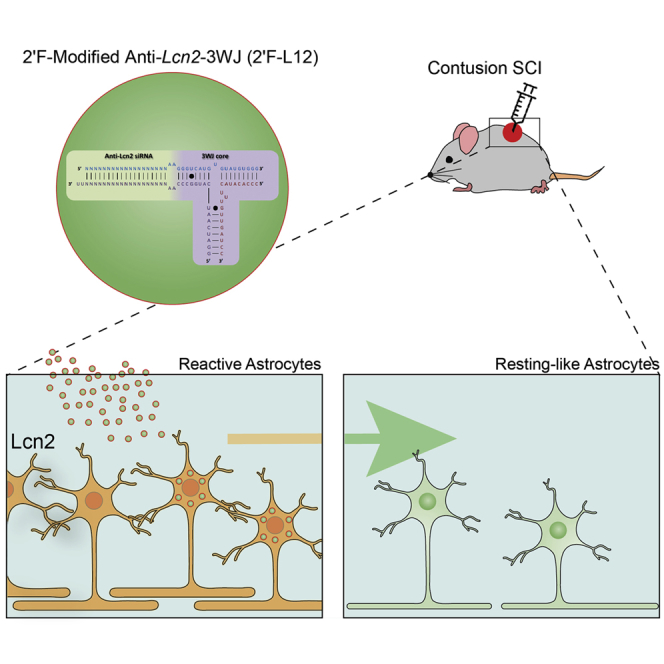

## Introduction

Astrocytes are abundant and highly heterogeneous cells that provide a variety of essential support and regulatory functions within the CNS.^1^, ^2^ In response to CNS pathologies such as spinal cord injury (SCI), stroke, multiple sclerosis (MS), and Alzheimer’s disease, astrocytes show changes in gene expression, cellular structure, and function. Such responses are commonly referred to as astrogliosis.^3^, ^4^, ^5^, ^6^ Astrocyte reactivity is a ubiquitous response to CNS damage mediated by a complex and insult-specific mixture of cytokines, factors, and cellular debris, released by CNS resident damaged cells, inflammatory infiltrates, and even reactive astrocytes themselves, at and around the site of the insult.^7^ The pathological role of astrogliosis is controversial,^8^ but is generally considered to be a double-edged sword, mitigating the acute stresses that develop immediately following a pathogenic event, but ultimately serving as an impediment to neural plasticity as the phenomenon persists.^9^ For example, the archetypical hallmark of astrogliosis is the overexpression of cytoskeletal intermediate filament (IF) proteins, notably glial fibrillary acidic protein (GFAP) and vimentin (Vim), contributing to the hypertrophy and entanglement of reactive astrocytes that contribute to produce a dense barrier serving as the primary basis of the glial scar.^6^ This physicochemical barrier provides a protective fortification against the infiltration of inflammatory agents during the early stages of a CNS insult, but becomes an obstacle to remyelination, neuronal regeneration, and overall neural plasticity as the scar persists beyond the initial effects of the injury. Furthermore, reactive astrocytes play an important and complex role in modulating neuroinflammation, eliciting a context-dependent array of pro-inflammatory or anti-inflammatory effects in the CNS via secreted mediators and interactions with resident immune cells.^10^ One particularly notable vector is the secretion of the iron-trafficking protein Lipocalin 2 (Lcn2), which is canonically associated with innate immune responses but also described to act as a neuroinflammatogen in many CNS pathologies, including SCI and MS.^11^, ^12^ Astrocyte-secreted Lcn2 is known to exacerbate neuroinflammation by inducing pro-inflammatory polarization in recipient astrocytes and microglia, even promoting cell death in neurons.^13^

Thus, modulation of astrocyte reactivity, or specific aspects thereof, is a particularly attractive therapeutic strategy in the treatment of a diversity of CNS disorders.

One approach that has demonstrated some promising results has involved interfering with the astrogliotic overexpression of the IF proteins GFAP and Vim, particularly prominent targets in reactive astrocytes. *In vitro*, decreased IF expression has been shown to yield a concomitant decrease in astrocyte reactivity and hypertrophy, with co-cultured neurons exhibiting enhanced survivability and increased neurite growth.^14^, ^15^, ^16^, ^17^ *In vivo*, IF depletion has been found to result in decreased scar density, increased axonal plasticity, and improved functional recovery in mouse models of SCI and other CNS insults.^15^, ^16^, ^18^, ^19^, ^20^ These *in vivo* results have typically been obtained using constitutive knockout animal models devoid of *Gfap* and/or *Vim*. Such animals have an impaired ability to undergo astrogliosis and are thus denied the early protective aspects of reactive astrocytes, making them more susceptible to exacerbating mechanical injury despite longer-term improvements in axonal outgrowth. Indeed, the therapeutic utility of the wholesale ablation of astrogliosis is controversial.^8^ Perhaps a more promising therapeutic option is the modulation of injury-aggravating inflammation through depletion of secreted factors, such as Lcn2. Knockout of *Lcn2* has been found to largely negate the pro-inflammatory polarization of activated astrocytes *in vitro* and *in vivo*,^21^, ^22^ yielding better histopathological outcomes in mouse models of stroke, demyelinating diseases, or SCI.^11^, ^23^, ^24^, ^25^ *Lcn2*-deficient (*Lcn2*^−/−^) mice display remarkable neuronal survival and myelin sparing after contusion SCI.^11^ Importantly, these changes are accompanied by significant reduction of inflammatory responses and improved locomotor recovery.^11^

Nevertheless, gene knockout is not a clinically viable option, nor is it necessarily desirable because most genes also serve important physiological functions and their constitutive deletion can have adverse effects. An ideal therapy would consist of a temporally and dose-controlled means by which to modulate astroglial reactivities, such as that afforded by RNAi. Lentiviral delivery of short hairpin RNAs has been successfully used to reduce astrocyte reactivity *in vitro*;^14^, ^18^ yet there are safety concerns with the therapeutic translation of viral vectors. As an alternative, small interfering RNA (siRNA) therapeutics possess great potential for the transient knockdown of gene expression. *In vivo* functional recovery in SCI rodents has been observed using *Gfap*- and *Vim*-targeting siRNAs;^15^, ^16^ however, the clinical translation of siRNA therapeutics has been hampered by their relative fragility and inefficiency in their delivery. Nevertheless, the burgeoning field of nanotechnology offers hope for the development of effective, targeted RNA-based therapeutics. Nanotechnological RNA delivery vehicles encompass a myriad of chemistries including complexation with carrier polymers, encapsulation within lipid vesicles, or coating onto organic or inorganic nanoparticles. Moreover, due to its flexibility and intrinsic potential for target recognition and enzymatic activity, RNA itself has been employed as a scaffold from which to develop multifunctional nanostructures.^26^, ^27^, ^28^ Building upon the principles of DNA origami^29^ and tecto-RNA,^30^ a variety of RNA-based nanotriangles,^31^ cubes,^32^ rings,^33^ and lattices^34^ have been designed as putative therapeutic platforms. Self-assembly of siRNA polymers generated via rolling circle transcription has even allowed for the generation of RNAi microsponges^35^ and nanosheets.^36^

Herein, we describe proof-of-concept evidence of the packaging RNA (pRNA) three-way junction (3WJ) as a platform for the delivery of anti-IF or anti-*Lcn2* siRNAs to reactive astrocytes *in vitro* and *in vivo*. The pRNA-3WJ, itself a basic unit for the construction of more elaborate RNA nanostructures,^37^ has demonstrated remarkable versatility as a targeting and therapeutic delivery agent, excelling in the targeted delivery of anti-miRNAs and siRNAs.^38^, ^39^, ^40^, ^41^ The pRNA platform is highly stable, non-toxic, biocompatible, and ideally sized for nanotherapeutic applications,^42^ with the multi-armed nature of the 3WJ core allowing for the design of multifunctional, modular constructs.^43^, ^44^ This technology has seen extensive use in anti-cancer or anti-viral roles, but here we report the first progress toward applications in the CNS niche.

## Results

### Astrocytes Cultured in Low-FBS Medium Enter a Reactive State When Exposed to Lipopolysaccharide + Interferon-γ

Differentiation of neural stem cells (NSCs) into astrocytes was induced by the presence of fetal bovine serum (FBS), a well-established technique,^45^, ^46^, ^47^ with 15 days *in vitro* (DIV) chosen as a time point at which cultured astroglial cells had achieved a desirable level of maturity.^47^ Immunofluorescence imaging revealed uniform expression of GFAP and Vim, and high expression of the astroglial markers S100 calcium-binding protein B (S100B)^48^ and 10-formyltetrahydrofolate dehydrogenase (Aldh1l1)^49^ (Figure S1). We employed a two-phase differentiation protocol, switching from high-FBS (10%) to low-FBS (1%) N2-supplemented medium at 7 DIV to minimize the intrinsic astroglial reactivity attributable to the described stimulatory effect of serum.^35^, ^36^ Differentiation medium was supplemented with FGF2 to further promote a mature, resting-like phenotype.^50^ By contrast to the hypertrophic, polygonal astrocyte clusters derived via the high-FBS technique, low-FBS astrocytes were confluent and ramified with long processes (Figure S1). These morphological differences, coupled with the significantly less intense GFAP and Vim expression observed in low-FBS astrocytes, suggested a strong basal reactivity in high-FBS astrocytes.

In order to have a simple and reproducible *in vitro* model of astrogliosis, we activated astrocytes through exposure to lipopolysaccharide (LPS) and interferon-γ (IFN-γ).^51^ Our refined activation protocol involved exposure to LPS+IFN-γ for 48 hr at 15–17 DIV, with RNA analyses at 17 DIV and protein analyses at 20 DIV.

**Figure 1:**
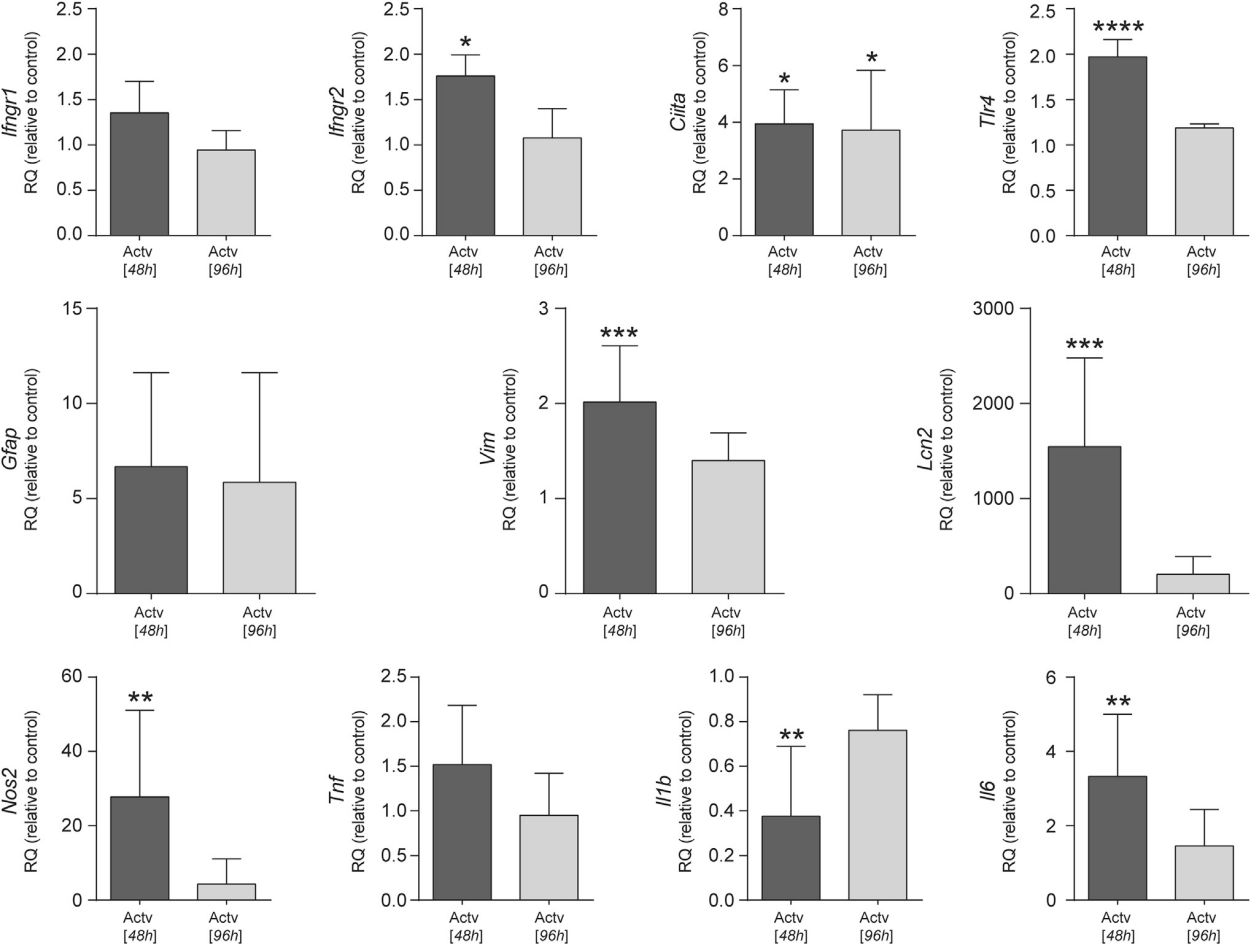
Response of Low-FBS/+FGF2 Cultured Astrocytes to LPS+IFN-γ Stimulation at the mRNA Level qRT-PCR quantification of relative mRNA expression levels of *Ifngr1*, *Ifngr2*, *Ciita*, *Tlr4*, *Gfap*, *Vim*, *Lcn2*, *Nos2*, *Tnf*, *Il1b*, and *Il6*. Expression relative to resting controls (2^−ΔΔCt^ method), *Gapdh* reference gene. Data expressed as the mean of n ≥ 3 biological replicates ± SD; *p ≤ 0.05, **p ≤ 0.01, ***p ≤ 0.001; or ****p ≤ 0.0001, relative to non-activated control samples (one-way ANOVA with Dunnett’s multiple comparison test).

Low-FBS astrocytes treated with LPS+IFN-γ for 48 hr exhibited an upregulation of Toll-like receptor 4 (*Tlr4*) and IFN-γ receptors 1 and 2 (*Ifngr1*/*Ifngr2*). After a subsequent 48 hr in LPS+IFN-γ-free medium, expression of the above mRNAs reverted to basal levels. Likewise, major histocompatibility complex class II transactivator (*Ciita*) was induced in the 48 hr following activation and persisted elevated at 96 hr (Figure 1). We also observed an increased expression of both *Gfap* and *Vim* at 48 hr post-treatment. However, the induction of *Gfap* failed to achieve statistical significance. Instead, *Lcn2* expression underwent a 15,000-fold induction upon 48-hr LPS+IFN-γ treatment, dropping off significantly after a further 48 hr in LPS+IFN-γ-free medium. Other accepted pro-inflammatory genes were upregulated after LPS+IFN-γ, including interleukin-6 (*Il6*), tumor necrosis factor (*Tnf*), and nitric oxide synthase 2 (*Nos2*). The expression of these activation genes also reverted toward resting levels upon withdrawal of the LPS+IFN-γ medium. Conversely, the expression of the pro-inflammatory cytokine *Il1b* was significantly downregulated by LPS+IFN-γ (Figure 1).

**Figure 2:**
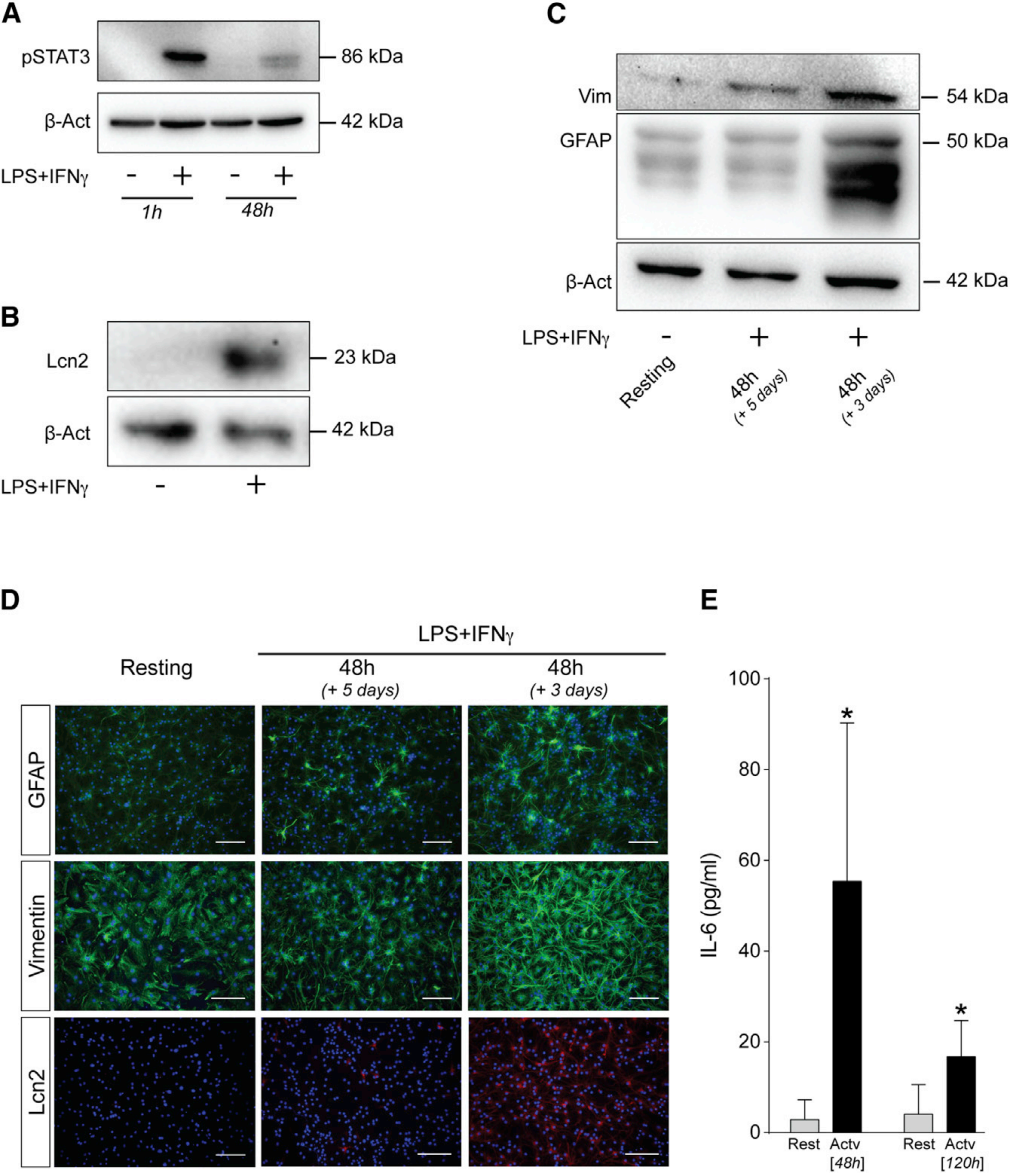
Response of Low-FBS/+FGF2 Cultured Astrocytes to LPS+IFN-γ Stimulation at the Protein Level (A) Representative western blot illustrating temporal induction of pSTAT3 upon 1 and 48 hr of treatment with LPS+IFN-γ. (B and C) Representative western blots of activation markers in resting versus LPS+IFN-γ-stimulated low-FBS astrocytes: (B) Lcn2 expression in resting and 24-hr-stimulated astrocytes; and (C) GFAP and Vim levels under resting conditions and following 48 hr of activation followed by 3 or 5 days of rest. β-Actin was used as a loading control. (D) Representative immunofluorescence micrographs of GFAP, Vim, and Lcn2 immunoreactivity in resting and activated conditions (GFAP/Vim: green; Lcn2: red; DAPI nuclear stain: blue). Scale bars, 100 μm. (E) Secreted IL-6 as measured by ELISA, resting versus activated conditions, at 48 and 120 hr post-stimulation. Data are expressed as the mean of n ≥ 3 biological replicates ± SD. *p ≤ 0.05, relative to non-activated control samples (multiple t tests with statistical significance determined using the Holm-Sidak method, α = 0.05).

We also examined the effects of the activation of low-FBS astrocytes at protein level, most fundamentally by an increase in pSTAT3 (Figure 2A). The kinetics of this induction were quite rapid, with pSTAT3 showing a peak in expression at 1 hr and subsequent decrease by 48 hr. At a more general level, activated astrocytes also exhibited increased early expression of Lcn2 (Figure 2B), as well as the IF proteins GFAP and Vim (Figure 2C), most evident at 3 days post-activation. In immunofluorescence assays, Lcn2 exhibited a remarkable upregulation, from no obvious expression in resting-like conditions up to an intense expression in most cells upon activation (Figure 2D). This activation response was further reflected by an approximately 50-fold increase in IL-6 secretion at 48 hr after LPS+IFN-γ (Figure 2E).

On the other hand, high-FBS astrocytes showed a constitutive high reactivity, evident from significantly higher basal expression of Vim, GFAP, and Lcn2, as compared with resting-like low-FBS astrocytes. Moreover, ostensibly resting-like high-FBS astrocytes were seen to have a strong pSTAT3 expression. High-FBS astrocytes treated with LPS+IFN-γ for 48 hr exhibited significant inductions in *Nos2* and *Il6*, as well as increased secretion of IL-6. However, *Tnf* expression showed no change, nor did *Gfap* and *Vim*. An induction of *Lcn2* was evident upon LPS+IFN-γ treatment. *Il1b* also remained unchanged upon activation; however, levels in the high-FBS astrocytes were significantly lower than low-FBS analogs (Figure S2).

These findings supported the use of low-FBS astrocytes for the experiments described hereafter.

### siRNA-3WJ Nanostructures Have Negligible Toxic Effects upon Transfection into Astrocytes

**Figure 3:**
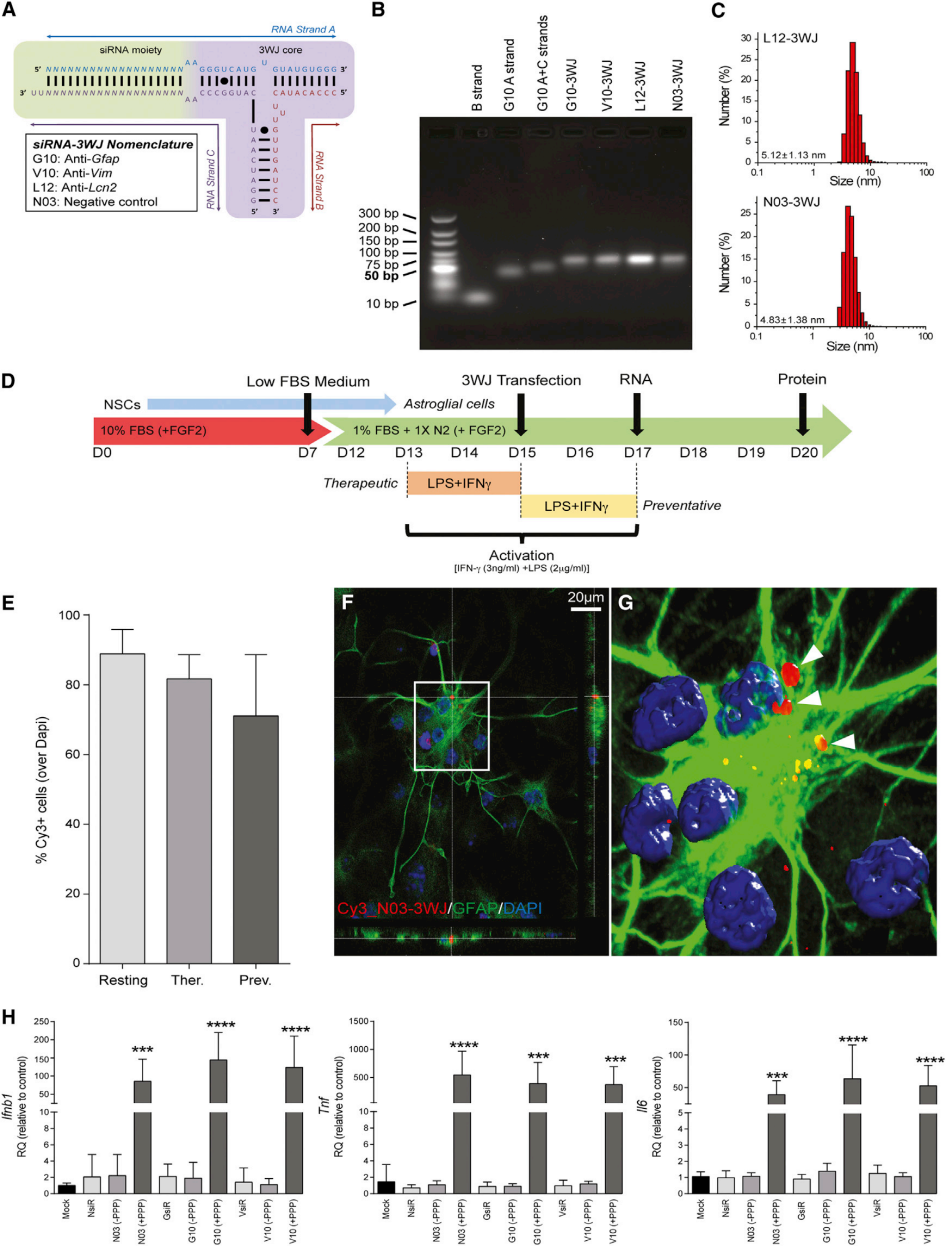
Synthesis, Delivery, and Safety of siRNA-3WJ Nanostructures (A) Generic 3WJ scheme depicting the three constituent RNA strands, variable siRNA (*N*) and 3WJ core moieties (black circles indicate a wobble base pair). (B) Agarose gel (2%, TBM buffer) illustrating the characterization of 3WJ assembly by gel mobility. The assembled 3WJs show retarded mobility relative to a double-stranded (*A*+*C* strands) construct or single *A* or *B* strands alone. An Ultra-Low Rage dsDNA ladder is used as reference. (C) Dynamic light scattering (DLS) size characterization of representative L12- and N03-3WJs. Results are expressed as mean hydrodynamic size (nm) ± SD. (D) Differentiation/treatment scheme: NSCs are differentiated in a medium containing high FBS for 7 days (red) before being swapped to a low-FBS medium supplemented with N2 (green) to obtain cells with a mature, resting astrocyte phenotype. Cells were transfected with 3WJs at day 15 (D15), with RNA and protein expressions were assayed at days 17 and 20, respectively, unless otherwise noted. Activation of astrocytes is achieved through the addition of LPS+IFN-γ to the medium for 48 hr: from D13 through D15 for the *therapeutic* treatment profile, and from D15 to D17 for the *preventative* profile. (E) Immunofluorescence quantification of percentage of astrocytes transfected with 20 nM Cy3_N03-3WJ under resting and activated conditions. Data are expressed as mean ± SD; n = 3 biological replicates, 3 coverslips per condition for each experiment, and 10 images per coverslip. No statistically significant difference was observed between conditions. (F) z stack confocal micrograph of astrocytes transfected with 20 nM Cy3_N03-3WJ (red), fixed 24 hr after transfection, and immunostained for GFAP (green), with nuclei co-stained with DAPI (blue). (G) Volocity-based 3D reconstruction (from a total of n = 22 z stacks of optical slices in 0.25-μm intervals) of the confocal z stack in (F). (H) Expression of pro-inflammatory cytokines (*Ifnb1, Tnf*, and *Il6*) in high-FBS/FGF2-free cultured “resting” astrocytes when transfected with 3WJs (with [+PPP] or without [−PPP] 5′-triphosphate *in vitro* transcription artifacts) or analogous commercial siRNA pools (NsiR, GsiR, or VsiR, being non-targeted, anti-*Gfap*, or anti-*Vim*, respectively). Results expressed as the mean mRNA expression (qPCR) relative to non-transfected controls (2^−ΔΔCt^ method) with n ≥ 3 biological replicates ± SD; *Gapdh* reference gene. *p ≤ 0.05; **p ≤ 0.01; ***p ≤ 0.001; or ****p ≤ 0.0001, relative to control samples (one-way ANOVA with Dunnett’s multiple comparison test).

siRNA-3WJs of the general scheme depicted in Figure 3A were prepared based on the methods described previously.^43^ The siRNA-3WJs used herein were named according to their target mRNA and the identification number of each given SMARTpool siRNA analog (Table S1), thus giving rise to G10-3WJ (anti-*GFAP*), V10-3WJ (anti-*Vim*), L12-3WJ (anti-*Lcn2*), and the non-targeted N03-3WJ (as negative control). The three component strands of each siRNA-3WJ were mixed in stoichiometric ratio and annealed to generate the 3WJ nanoparticle. Successful assembly of the siRNA-3WJs was confirmed by the decreased gel mobility of the multistranded structures in electrophoretic assays relative to single- and double-component constructs (Figure 3B), with dynamic light scattering measurements sizing the siRNA-functionalized L12- and N03-3WJs at 5.12 ± 1.13 and 4.83 ± 1.38 nm, respectively (Figure 3C).

Once assembled, siRNA-3WJs were delivered to astrocytes *in vitro* by means of lipofection. Unless otherwise noted, transfections occurred at 15 DIV with subsequent evaluation of mRNA levels via qPCR at 17 DIV and proteins via western blot or fluorescence microscopy at 20 DIV. Transfections were performed in three different activation contexts: a *resting* profile in which astrocytes were not treated with LPS+IFN-γ, and two different activation profiles wherein LPS+IFN-γ treatment occurs either in the 48 hr prior to transfection (i.e., 13–15 DIV) or the 48 hr commencing with transfection (i.e., 15–17 DIV) (Figure 3D). The former activation profile, henceforth referred to as a *therapeutic* intervention, simulates the treatment of an established CNS insult, whereas the latter profile, a *preventative* intervention, explores the utility of these putative nanotherapeutics in inhibiting a reactive response.

Transfection efficiency of siRNA-3WJs under these various activation conditions was assessed using Cy3-labeled N03-3WJ (Cy3_N03-3WJ) at a 20 nM concentration. As early as 24 hr post-transfection cells were fixed and processed for immunofluorescence, with the fraction of transfected astrocytes estimated by co-localization of punctuate Cy3 staining with GFAP-immunoreactive cells (over total nuclei). Transfection efficiencies, ascertained as the fraction of Cy3^+^ cells, were always found to be >70% under *resting*, *therapeutic*, and *preventative* conditions (Figure 3E). No uptake was evident without lipofection. Effective internalization of siRNA-3WJs was confirmed by confocal microscopy, with cross-sectional z stack images of Cy3_N03-3WJ transfected low-FBS astrocytes showing the presence of Cy3^+^ aggregates within the cell bodies (Figures 3F and 3G).

Transfection of astrocytes with these siRNA-3WJ constructs proved to be non-toxic over the range of concentrations tested. Using colorimetric LDH cytotoxicity assays, we found that the viability of cells remained >95% for all siRNA-3WJs (N03-, G10-, V10-, and L12-) and concentrations tested, thus demonstrating no significant cytotoxicity relative to untreated controls or cells treated with vehicle only. No toxicity was observed at 24 hr post-transfection, when astrocytes remained in serum-free transfection media, or as late as 120 hr after transfection (Figure S3). Furthermore, we checked the stability of several common reference genes, including glyceraldehyde-3-phosphate dehydrogenase (*Gapdh*), 18S ribosomal RNA (*Rna18s5*), and beta-actin (*Actb*), via TaqMan gene expression assays to establish their suitability in subsequent qPCR assays. The expression of each of these putative reference genes at 48 hr post-transfection showed no variation across treatment or condition (Figure S4).

The potential immunogenicity of the siRNA-3WJs was tested with respect to their ability to induce the expression of pro-inflammatory cytokine genes in astrocytes. Preliminary assays utilized 3WJs assembled from *in vitro* transcribed ***A*** and ***C*** strand single-stranded RNAs (ssRNAs) that had not been subjected to de-phosphorylation and thus still possessed 5′-triphosphate transcription artifacts. Transfections with 5′-triphosphate N03-, G10-, and V10-3WJs (5 nM) were found to cause significant inductions in *Il6* (∼50-fold), *Tnf* (∼400-fold), and IFN beta 1 (*Ifnb1*, ∼100-fold) relative to non-transfected controls (Figure 3H). Because neither mock-transfected samples nor those treated with commercial siRNA pools (chemically synthesized and with proprietary modification) had such an effect, we hypothesized this unwanted exacerbation of the pro-inflammatory response to be the result of retinoic acid-induced gene 1 (RIG-I)-mediated antiviral immunity,^52^ triggered by the 5′-triphosphate transcription artifacts. Indeed, introduction of an RNA dephosphorylation step into the 3WJ synthesis completely abolished the induction of IL-6 and TNF-α, at both mRNA (Figure 3H) and protein levels (Figure S3).

Thus, appropriately modified 3WJs were found to be non-immunogenic.

### siRNA-3WJ Nanostructures Efficiently Downregulate the Expression of Reactive Astrocyte-Associated Genes *In Vitro*

Preliminary assays in high-FBS astrocytes revealed a dose-dependent silencing response by G10- or V10-3WJs. By qPCR assays, knockdown of target genes was found to be >50% at 5 nM relative to controls, with no appreciable off-target silencing effects (Figure S5). Commercial siRNA pools containing multiple chemically modified siRNAs against the target gene yielded results comparable with the analogous unmodified siRNA-3WJs bearing a single siRNA against the same target (Figure S5). Higher concentrations (i.e., 50 nM) revealed no further increase in efficacy (data not shown).

Having established 5 nM of either IF-targeted siRNA-3WJ as being sufficient to induce a significant reduction in each respective mRNA, this concentration was used in subsequent experiments to further examine the utility of the treatments under activation conditions, as well as to investigate siRNA-3WJ-mediated *Lcn2* knockdown.

**Figure 4:**
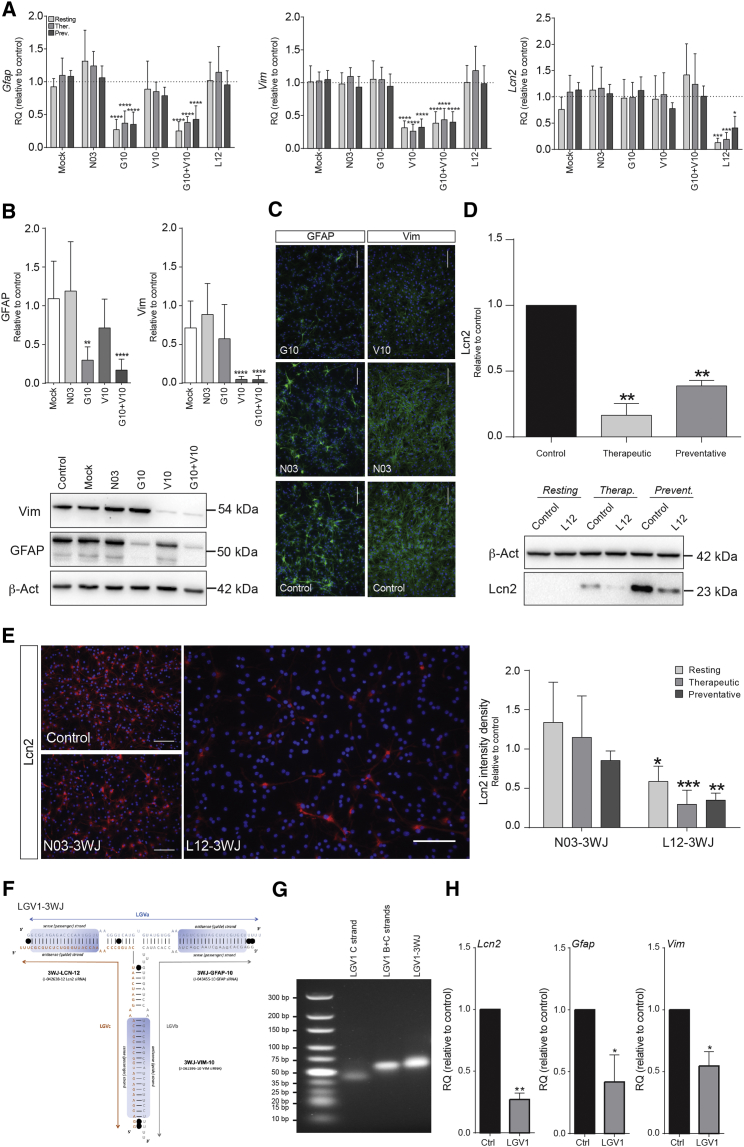
siRNA-3WJ Nanotherapeutics Specifically and Significantly Knock Down Expression of Target Genes (A) qPCR assays showing the knockdown of *Gfap*, *Vim*, and *Lcn2* target mRNA by 5 nM doses of their respective siRNA-3WJ nanostructures. Downregulation measured 48 hr after transfection, under resting conditions. Mock (Lipofectamine-only) or non-targeted negative control 3WJs were used as negative controls. *Gapdh* was the reference gene. (B) Densitometric quantification of Vim (54 kDa) and GFAP (50 kDa) western blots showing siRNA-3WJ-induced protein knockdown in *preventative* activation conditions (20 DIV) and a representative image. Quantification is expressed relative to β-actin and normalized to non-transfected control samples. (C) Representative immunofluorescence micrographs depicting the decrease in GFAP and Vim immunoreactivity upon G10- or V10-3WJ treatment, respectively, in *preventative* activation conditions, compared with non-treated and N03-3WJ-treated controls (GFAP and Vim: green, DAPI-stained nuclei: blue). (D) Quantification and representative image of Lcn2 (23 kDa) western blot under *resting*, *therapeutic*, and *preventative* activation conditions upon L12-3WJ treatment (16 DIV), compared with non-treated controls. Quantification as per (B). (E) Lcn2 immunofluorescence, as per (C), with quantification of intensity density (n = 3 biological replicates, 2 coverslips per replicate, 10 fields of view per coverslip) of L12- and N03-3WJ-treated samples versus non-treated controls (Lcn2: red; DAPI-stained nuclei: blue). Scale bars, 100 μm. (F) Schematic of the LGV1-3WJ, a single 3WJ core functionalized with the siRNA moieties of each of the L12-, G10-, and V10-3WJs. *In vitro* transcribed RNA strands LGVa, LGVb, and LGVc are annealed together; black circles indicate a wobble base pair. (G) Agarose gel (3%, TBM buffer) illustrating the stepwise assembly and retarded mobility of the extended LGV1-3WJ construct relative to single- and double-stranded intermediates. Ultra-low dsDNA ladder is for reference. (H) qPCR assays showing the knockdown of *Gfap*, *Vim*, and *Lcn2* target mRNA by a 5 nM dose of the LGV1-3WJ nanostructure, as per the single-siRNA 3WJs in (A). Quantitative results expressed as the mean of n ≥ 3 biological replicates ± SD. *p ≤ 0.05; **p ≤ 0.01; ***p ≤ 0.001; or ****p ≤ 0.0001, relative to control samples of the same activation condition (two-way ANOVA [qPCR] or one-way ANOVA [western blot] with Dunnett’s multiple comparison test).

In low-FBS astrocytes, we achieved significant reductions of *Gfap* and *Vim* levels by G10- and V10-3WJs, both in resting and LPS+IFN-γ-activated cells, under *therapeutic* and *preventative* settings. In both resting and activation conditions, G10-3WJ decreased *Gfap* expression by >60% (Figure 4A). V10-3WJs yielded similar results, with *Vim* expression knocked down by >65% (Figure 4A). No appreciable synergistic effects were evident in combinatorial treatments comprising both G10 and V10 simultaneously (Figure 4A).

Knockdown of Lcn2 by L12-3WJs was consistent with the absolute expression levels of *Lcn2* in resting versus activated astrocytes, with the most significant knockdown (87.1% ± 3.7%) being observed in the resting condition (Figure 4A). L12-3WJ treatments in *therapeutic* and *preventative* conditions exhibited somewhat lower knockdowns relative to their non-treated controls (81.1% ± 5.4% and 59.2% ± 9.8%, respectively) (Figure 4A). 2′-Fluoro-modified L12-3WJ, intended for *in vivo* use due to the higher nuclease resistance of the 2′F modification,^53^ demonstrated a comparable knockdown efficacy versus the 2′-OH form (63.7% ± 4.9%) (Figure S6). LGV1-3WJ, bearing all three of the siRNA moieties possessed by the L12-, G10-, and V10-3WJs, produced significant downregulation of each of the three target mRNAs simultaneously at a 5 nM dose under *preventative* conditions (Figures 4F–4H).

No significant off-target effects were ever observed for targeted 3WJs, nor did the non-targeted N03-3WJ induce any significant change in the expression of *Gfap*, *Vim*, or *Lcn2* (Figure 4A). *Gfap-*, *Vim-*, or *Lcn2-*targeted 3WJs failed to affect the expression of pro-inflammatory cytokine genes that included *Il6*, *Nos2*, *Tnf*, and *Il1b* (Figure S7).

In activated astrocytes, both G10- and V10-3WJs induced significant reductions in GFAP and Vim protein expression, respectively (Figures 4B and 4C). GFAP levels were decreased 70.3% ± 7.0% by G10-3WJ treatment, whereas Vim was down 95.0% ± 1.7% upon V10-3WJ treatment, both in the *preventative* setting. Combined treatments invoked comparable downregulation in protein expression for both IFs, ruling out synergistic effects. A further qualitative assessment of GFAP or Vim expression by immunofluorescence confirmed the results of the western blots (Figure 4C).

Lcn2 was detected in activated astrocytes by western blot at both 16 DIV (*therapeutic* and *preventative*) and 17 DIV (*preventative* only) (Figure 4D). Treatment with L12-3WJ yielded a significant reduction of Lcn2 in each condition (Figure 4D): 83.6% ± 5.1% reduction for the *therapeutic* setting and 61.2% ± 2.4% for the *preventative*. This striking reduction in Lcn2 expression was also evident by immunofluorescence, with *therapeutic* and *preventative* samples exhibiting Lcn2 immunofluorescence reductions of 67.4% ± 7.0% and 57.8% ± 5.0%, respectively (Figure 4E).

Thus, siRNA-3WJs nanostructures can efficiently downregulate the expression of reactive astrocyte-associated mRNAs and proteins.

### siRNA-3WJ Nanostructures Downregulate Lcn2 in Reactive Astrocytes *In Vitro* to Inhibit the Propagation of Astroglial Responses

**Figure 5:**
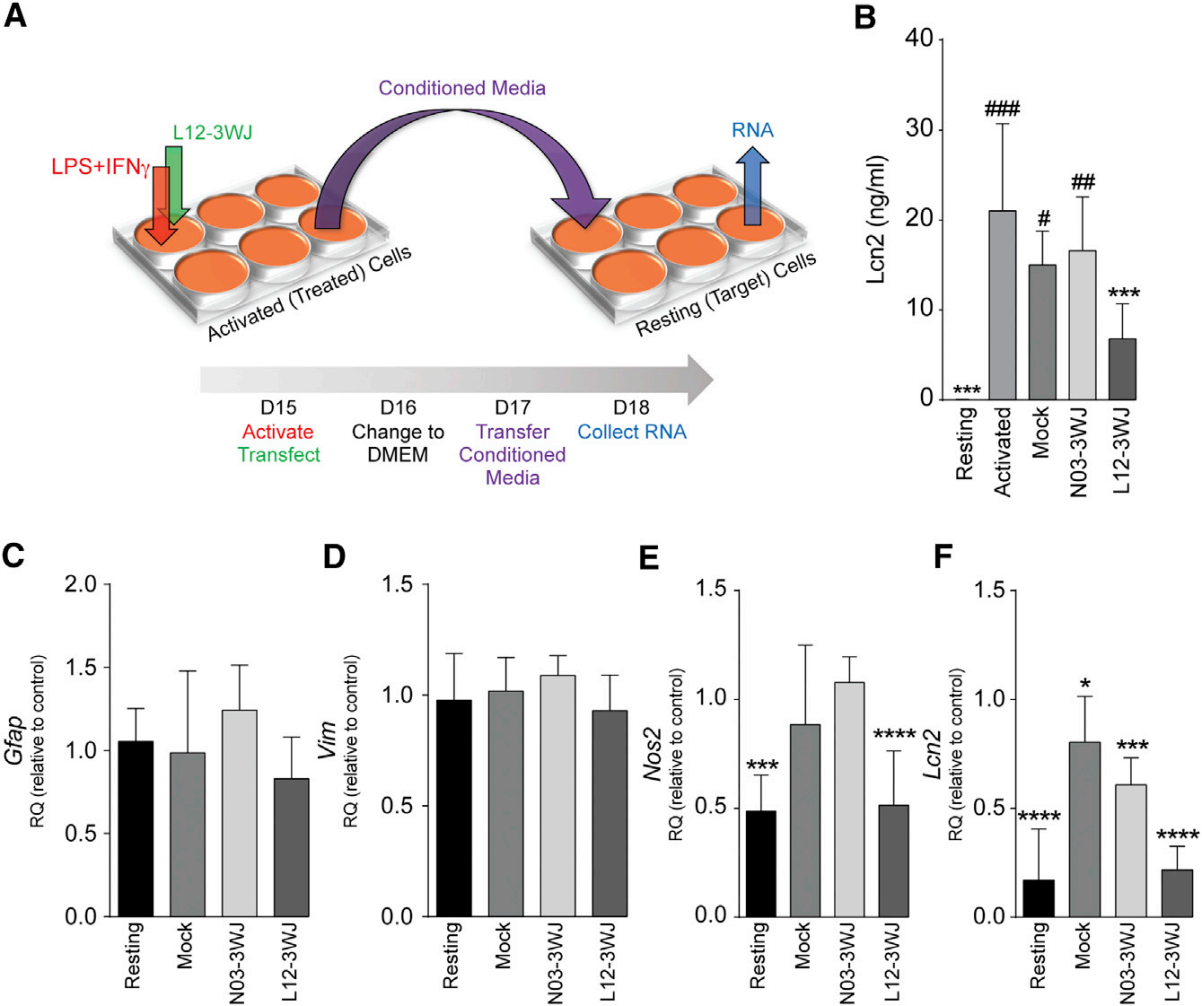
L12-3WJ Treatment Ameliorates the Propagation of Classical Activation by Reactive Astrocytes (A) Schematic of the conditioned media transfer experiment. (B) ELISA quantification of Lcn2 in the supernatant media of resting or LPS+IFN-γ-stimulated astrocytes, as well as activated astrocytes treated with mock, N03, or L12 transfections, 48 hr after treatment. Asterisk (*) denotes statistical significance relative to the supernatant of non-transfected, activated astrocytes; number sign (#) is relative to non-transfected, resting astrocytes. ##p ≤ 0.01, ###p ≤ 0.001 relative to resting medium, ***p ≤ 0.001 relative to activated medium. (C–F) qPCR quantification of activation-associated genes in recipient cells 24 hr after transfer of media from treated astrocytes: (C) *Gfap*, (D) *Vim*, (E) *Nos2*, and (F) *Lcn2*; results are expressed as the mean mRNA expression relative to those cells receiving conditioned media from activated controls (2^−ΔΔCt^ method) (*Gapdh* reference gene). Data represent the mean of n ≥ 3 biological replicates ± SD. *p ≤ 0.05; **p ≤ 0.01; ***p ≤ 0.001; or ****p ≤ 0.0001, relative to control samples in the same activation condition (two-way ANOVA with Dunnett’s multiple comparison test).

Secretion of Lcn2 significantly increased in activated astrocytes. Replacing cell media with fresh DMEM 24 hr after activation and subsequently conditioning that DMEM for an additional 24 hr yielded secreted Lcn2 concentrations of 0.2 ± 0.1 ng/mL in resting astrocytes and 21.0 ± 3.2 ng/mL in activated astrocytes (Figure 5B). Concurrent treatment of activated astrocytes with L12-3WJ reduced the mean secreted Lcn2 concentration to 6.8 ± 1.3 ng/mL, whereas mock or N03-3WJ treatments had no effect.

To investigate the capacity of siRNA-3WJ nanostructures to inhibit the propagation of astroglial responses, we assessed the effects of activated astrocyte-conditioned media (aACM) on resting (second-generation) astrocytes (as per the scheme in Figure 5A). *Gfap* (Figure 5C) and *Vim* (Figure 5D) expression levels were unchanged by aACM, with no appreciable induction between astrocytes treated with aACM and control ACM. Conversely, we observed a significant increase in the expression of *Nos2* (Figure 5E) and *Lcn2* (Figure 5F) in astrocytes receiving aACM (2.1-fold and 5.9-fold, respectively). Interestingly, treatment of first-generation astrocytes with L12-3WJ significantly reduced the induction of both *Nos2* and *Lcn2* in aACM-treated second-generation astrocytes (Figures 5E and 5F). *Lcn2* also appeared downregulated, albeit much less significantly, by treatment with the ACM from those astrocytes mock-transfected or transfected with the non-targeted control N03-3WJ (Figure 5F).

### siRNA-3WJ Nanostructures Downregulate Lcn2 *In Vivo* in an Experimental Model of Moderate Contusive SCI

**Figure 6:**
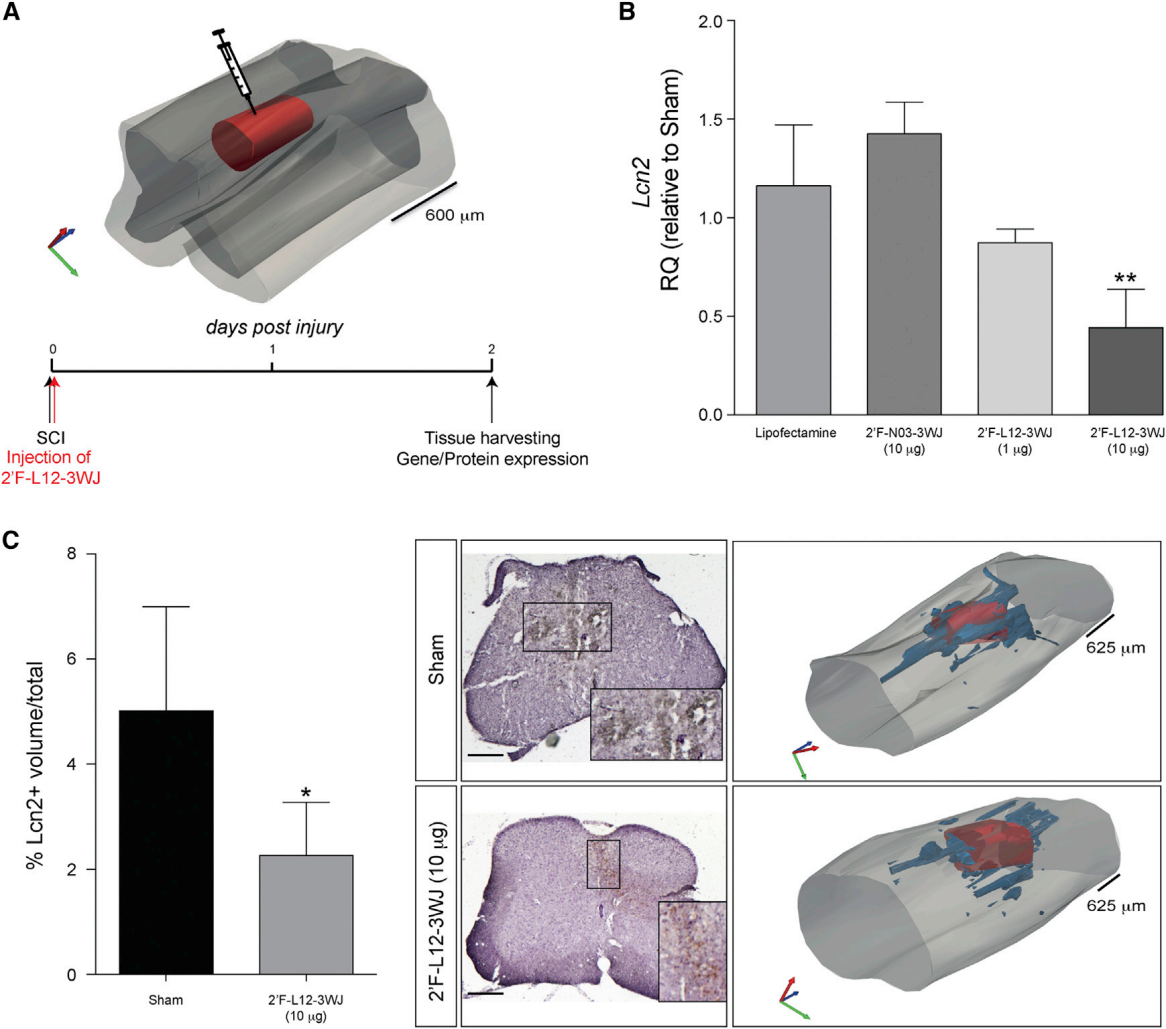
2′F-L12-3WJ Injection in an Experimental Model of Moderate Contusive Spinal Cord Injury Reduces Lcn2 mRNA and Protein Levels (A) Schematic representation of 2′F-L12-3WJ injection in an experimental model of moderate contusive spinal cord injury. (B) qPCR quantification showing the knockdown of Lcn2 in mice treated with 10 μg of 2′F-L12-3WJ, compared with injury-only control mice (sham), Lipofectamine-only treated mice, 10 μg of 2′F-N03-3WJ, and 1 μg of 2′F-L12-3WJ-treated mice (n = 3–4 mice per group). **p ≤ 0.01, relative to sham control mice (one-way ANOVA with Dunnett’s multiple comparison test). Data represent mean ± SD. (C) Quantification of Lcn2-postive volumes in injury-only control mice (sham) versus 10 μg of 2′F-L12-3WJ-treated mice. Data are expressed as % of Lcn2^+^ volume ± SD over the total volume (n = 4 mice per group). *p ≤ 0.05 (Student’s t test). Representative Lcn2 immunohistochemistry images in injury-only control (sham) and 10 μg of 2′F-L12-3WJ-treated mice, counterstained with hematoxylin. Original magnification ×4; scale bar, 500 μm. Corresponding representative 3D reconstructions depict the lesion volume in red, with blue contours representing Lcn2^+^ volumes and light gray contour representing the spinal cord.

To determine the efficacy of L12-3WJ administration *in vivo*, we employed an experimental model of moderate contusive SCI. It has been observed, both in our own studies (data not shown) and in the literature,^11^ that Lcn2 expression is significantly upregulated at the mRNA and protein levels starting 1 day postinjury, mainly in activated astrocytes and infiltrating leukocytes. After performing moderate injury, 2′F-L12-3WJs or control 3WJs were injected into the lesion epicenter and mice were sacrificed after 2 days (Figure 6A). These preliminary *in vivo* data showed a significant reduction (55.7% ± 9.8%) of *Lcn2* mRNA in SCI mice treated with a 10 μg dose of 2′F-L12-3WJ, compared with SCI mice injected with control treatments, whereas SCI mice treated with a 1 μg dose of 2′F-L12-3WJ showed little reduction in mRNA expression (Figure 6B). Significant Lcn2 reduction in 2′F-L12-3WJ-treated mice was observed at the protein level via immunohistochemical staining, with quantification of Lcn2^+^ volumes in these treated mice showing an approximately 55% reduction in Lcn2 expression compared with sham-treated control SCI mice (Figure 6C).

These preliminary *in vivo* data indicate that the local delivery of L12-3WJ can efficiently downregulate the expression of our gene of interest at both mRNA and protein levels.

## Discussion

Astrocytes are a heterogeneous population of cells, which play significant roles in a multitude of functions integral to the maintenance of CNS homeostasis. In response to a diversity of diseases or injuries, astrocytes enter a reactive state marked by changes in gene expression, function, and morphology.^3^, ^4^, ^54^ This phenomenon, known as astrogliosis, is a complex and heterogeneous response to molecular stimuli that falls on a continuum of severity depending on the context, magnitude, or proximity of the insult.^10^, ^55^ Indeed, healthy resting astrocytes exhibit varying degrees of reactivity in the normal physiological function of the CNS, evincing the non-binary nature of activation. A canonical manifestation of astrogliosis is the overexpression of IF proteins such as GFAP and Vim, with subsequent hypertrophy and entanglement of reactive astrocytes. In severe cases, astrocytes become proliferative and, in a complex interplay between multiple cell types and the extracellular matrix, form a dense glial scar.

Several *in vitro* and *in vivo* studies of reactive astrocytes show modulation of a plethora of different structural and functional molecules in an insult-specific and temporally dependent manner. This diversity is reflected in the profile of effector molecules secreted by reactive astrocytes, pro-inflammatory and anti-inflammatory cytokines, growth factors, and inhibitory agents, and thus the influence of such astrocytes on their environment. Consequently, astrogliosis is seen as a double-edged sword in the context of CNS afflictions such as SCI, where reactive astrocytes can play both beneficial and detrimental roles. Thus, from a therapeutic perspective, gene knockout approaches toward ablating astrogliosis are undesirable because they also inhibit the *beneficial* aspects of acute astrocyte reactivity and other likely physiological roles for the gene of interest, in addition to being clinically infeasible. A more translational approach could be a controlled temporal regulation of astrogliosis, focusing on chronically or excessively reactive astrocytes and/or specific deleterious aspects of astrogliosis, rather than complete suppression of the response.

Lcn2 is emerging as one such promising therapeutic target because of its significant role in astrogliosis and neuroinflammation.^13^ Ostensibly involved in iron regulation and the innate immune response, Lcn2 is upregulated/secreted by reactive astrocytes^21^, ^22^, ^56^, ^57^, ^58^, ^59^ and has been implicated in the modulation of inflammation in a number of CNS disorders, including SCI,^11^ MS,^25^, ^60^, ^61^, ^62^ and Parkinson’s disease.^63^ Autocrine/paracrine exposure to Lcn2 has been found to induce polarization toward a classically pro-inflammatory reactive phenotype in both astrocytes and macrophages, with typical GFAP upregulation and morphological changes in the former case, as well as sensitizing astrocytes and neurons to cytotoxicity.^22^, ^57^, ^58^, ^59^, ^64^ Knockout of astrocytic Lcn2 has been shown to ameliorate reactivity under *in vitro* and *in vivo* conditions in mouse neuroinflammation models, with Lcn2 deficiency found to attenuate the neurotoxic effects of LPS+IFN-γ treatment, inhibiting the overexpression of GFAP and pro-inflammatory markers.^11^, ^21^, ^24^ Moreover, Lcn2 knockout mice have exhibited improved neuronal and tissue survival, greater sparing of myelin, and significantly better locomotor recovery than wild-type mice following contusion SCI.^11^

Nevertheless, as with the IF proteins, knockout of the gene is not yet a clinically viable approach due to practical limitations and potential unintended effects (e.g., Lcn2 is believed to play roles in cognitive behavior and neurogenesis,^12^, ^65^ whereas, more broadly, inflammatory processes can convey some degree of neuroprotection under the right circumstances^66^).

Viral vectors are powerful tools for use in gene therapy applications and are regularly exploited for transient gene regulation via the delivery of RNAi agents. However, clinical translation of such systems is often hindered by several significant limitations, including the potential for immunogenicity or mutagenesis, or difficulties in production and scalability.^67^ An easily synthesized, modular, dosable, and non-toxic/non-immunogenic RNAi delivery platform, more reminiscent of a conventional pharmaceutical drug, has *potentially* lower hurdles to surmount on the path to clinical translation. Toward such a goal, we undertook proof-of-concept experiments to investigate the utility of pRNA-derived siRNA-3WJ nanotherapeutics in the CNS niche via downregulation of reactive astrocyte-associated genes (*Gfap*, *Vim*, and *Lcn2*) in an *in vitro* model of astrogliosis.

In establishing this model, we employed a convenient system by which to readily obtain activation-inducible astrocytes through the differentiation of NSCs^45^ in low-FBS, FGF2-supplemented media. The self-renewability of NSCs coupled with the ease of differentiation made this a more efficient option than harvesting primary astrocytes and provided a more homogeneous culture. Low FBS proved integral to obtaining confluent, basally resting astrocytes. We found the high-FBS (FGF2-free) cultured astrocytes to have a high basal expression of pro-inflammatory markers, IFs, and the activation marker pSTAT3. Such astrocytes were largely immune to subsequent canonical activation. Serum is known to invoke a reactive state in astrocytes,^68^, ^69^, ^70^ and FGF signaling has been found to suppress astrocyte activation^71^, ^72^ and generate a mature, quiescent phenotype with low GFAP expression in the astroglial differentiation of stem cells.^50^ Serum restriction and FGF2 supplementation resulted in more confluent cultures with a less-reactive morphology and phenotype, and a susceptibility to an inducible activation response.

Upon LPS+IFN-γ exposure, low-FBS cultured astrocytes showed evidence of both a classical pro-inflammatory response *and* canonical astrocyte reactivity. Increases in the mRNA expression levels of *Tlr4* and *Ifngr1/2* in the 48 hr following stimulation demonstrate the reactivity of these astrocytes to this activation protocol, with inductions in receptor expression in response to activation being previously reported in astrocytes for both *Tlr4*^73^, ^74^, ^75^ and *Ifngr*.^76^ Responsiveness to LPS+IFN-γ treatment was further evidenced by an increase in *Ciita*, a transcriptional co-activator of the major histocompatibility complex (MHC) class II, indicative of the acquisition of a non-professional antigen-presenting cell functionality in response to adaptive immune stimuli.^77^, ^78^, ^79^, ^80^ Furthermore, there was a significant induction in *Nos2* upon stimulation, an important indicator of activation and a precursor to the upregulation of IFs.^78^, ^81^, ^82^ This pro-inflammatory activation response was further evident here as a significant induction in the expression of *Il6* in LPS+IFN-γ-treated astrocytes. Absolute mRNA quantities of *Il6* and other examined cytokines were low (and thus inherently noisy) at 48 hr, possibly due to peak induction having occurred within 24 hr of activation^83^ and potentially masking relative changes in expression. Nevertheless, ELISA measurements performed on the supernatant of astrocytes activated for 48 hr did show a significant increase in the concentration of secreted IL-6. A transient LPS+IFN-γ-induced activation was observed by pSTAT3 western blots, peaking after just 1 hr of LPS+IFN-γ exposure. Lcn2, also regulated via STAT3 activation, was very significantly upregulated relatively quickly in LPS+IFN-γ-treated astrocytes. Intriguingly, *Il1b* mRNA levels were observed to *decrease* substantially upon LPS+IFN-γ stimulation despite IL-1β upregulation being commonly associated with reactive astrocytes. This might be attributable to an inhibitory effect of IFN-γ on IL-1β production that has previously been reported in mouse (but not human) astrocytes.^84^ This is supported by *in vivo* evidence that shows no IL-1 (α or β) induction in rat astrocytes upon intra-hippocampal LPS+IFN-γ injection.^85^ Similarly, the very low levels of IL-1β in high-FBS culture astrocytes might be because of their high basal expression of IL-6, which can also reportedly inhibit IL-1β expression.^86^

Low-FBS astrocytes also responded to LPS+IFN-γ-mediated activation by upregulating the expression of GFAP and Vim at both mRNA and protein levels. *Gfap* and *Vim* were notably higher than resting controls in the 48 hr post-activation, whereas at 96 hr post-activation the expression of both IF genes had reduced back to a level intermediate between their resting and 48 hr activated state. Protein levels quantified from western blots performed 5 days post-activation were likewise elevated, and increased immunoreactivity was also evident in fluorescence micrographs. This response, reminiscent of canonical astrogliosis, was ideally suited to subsequent characterization of siRNA-3WJ efficacy.

Our basic siRNA-3WJ designs were based on the pRNA-derived 3WJ architecture, well established as a novel and efficacious RNAi delivery platform.^43^ The multi-armed nature of the system makes it well suited to modular and/or multifunctional applications, with room for expansion beyond these current proof-of-concept experiments. Furthermore, the size of such siRNA-3WJs has previously been established to be approximately 5–30 nm (functionality dependent),^27^ optimally sized to be taken up into cells without significant loss to renal filtration or stimulation of the innate immune response. Moreover, this size is well suited to diffusion within the brain, being at the lower end of the range estimated for the size of the extracellular space.^87^ The pRNA system has demonstrated considerable promise in the targeted delivery of RNAi or small-molecule drugs for anti-cancer and anti-viral applications *in vitro* and *in vivo*,^26^, ^88^, ^89^ but these current studies represent the first applications in a CNS-specific environment. The present designs incorporate a single siRNA sequence per structure, each of which were selected to match the target sequences employed in commercially validated anti-*Gfap*, -*Vim*, -*Lcn2*, and non-targeting siRNA pools. Upon reaching an intracellular environment, the siRNA moiety is cleaved from the 3WJ by the endonuclease Dicer before entering the RNA-induced silencing complex (RISC) and guiding the cleavage of mRNA strands complementary to the target sequence.^90^

The two longer RNA strands of each 3WJ (***A*** and ***C*** strands) were prepared via *in vitro* transcription and dephosphorylated to remove 5′-triphosphate groups, an artifact of transcription using nucleotide triphosphates. In mammalian cells, the presence of uncapped 5′-triphosphate (or diphosphate) groups on nucleic acids is a known PAMP, recognized in the cytosol by the RIG-I helicase as a probable viral infection and triggering a type I IFN inflammatory response.^91^, ^92^ Indeed, siRNA-3WJs retaining their 5′-triphosphate groups provoked a significant pro-inflammatory response in transfected astrocytes, evident as increased levels of *Ifnb1*, *Il6*, and *Tnf*. This effect was mirrored by significant increases in the quantity of secreted IL-6 and TNF-α upon transfection with phosphorylated siRNA-3WJs. No such induction, neither at the mRNA nor protein levels, was evident upon transfection with the dephosphorylated constructs, which bypass the RIG-I-mediated antiviral response.^93^ An avoidance of RIG-I-mediated IFN responses is an important consideration for nucleic acid nanotherapeutics^94^ and particularly pertinent in this context because the activation of RIG-like receptor signaling has been directly implicated in the onset of astrogliosis.^95^ The dephosphorylated transcripts were subsequently annealed together with a third, synthetic ***B*** strand to yield the assembled construct.

Moreover, 3WJ transfection was found to have no off-target effect on the expression of putative reference genes in qPCR experiments, an important validation for experiments utilizing the 2^−ΔΔCt^ method of assaying relative gene expression.^96^ Our reference gene of choice, *Gapdh*, was unaffected by activation or transfection, with or without a 3WJ payload. 3WJs were found to be non-toxic, with doses as high as 50 nM showing negligible impact on the viability of astrocytes for at least 120 hr post-transfection.

Thus, these preliminary assessments speak to the safety of the 3WJ therapeutic platform in a CNS context; however, more extensive profiling, especially pertaining to potential off-target effects,^97^ would be required before any translational push.

Upon transfection, the siRNA-3WJs were successfully able to knock down the expression of their target genes in a specific and dose-dependent manner. Preliminary studies pointed toward an optimal dose of 5 nM as the basis for further investigation; higher doses yielded no appreciable increase in silencing efficacy, possibly because of saturation of the RNAi machinery.^98^ At this dose, the G10- and V10-3WJs induced >60% knockdown of their respective target genes relative to non-transfected controls. The extent of downregulation was similar under resting conditions and two different activation profiles: *preventative* and *therapeutic*. The *preventative* approach is intended to forestall the onset of activation, whereas the *therapeutic* method is perhaps the more clinically relevant scenario as it seeks to ameliorate already present astrogliosis. Combinatorial approaches employing both G10- and V10-3WJ did not reveal any synergistic, antagonistic, or compensatory effects, and neither anti-IF 3WJ had a significant effect on the expression of the other IF, at least at the mRNA level, despite the interfunctionality of these two IFs.^99^ Alternatively, treatment with LGV1-3WJ, targeting all three mRNAs, significantly downregulated each gene simultaneously, demonstrating the potential for multifunctionality and adaptability in the 3WJ platform. Mock and non-targeted N03-3WJ control transfections also had no effect on *Gfap* or *Vim* levels, ruling out any obvious side effects of lipofection, at least with regard to IF expression. L12-3WJ was found to nearly completely ablate the expression of *Lcn2* in resting astrocytes, again with no obvious effect on the other target genes, but was also highly efficient in activated astrocytes wherein *Lcn2* expression is many orders of magnitude higher. Such results are promising given the relatively low concentration of the siRNA-3WJs employed compared with other studies^15^ and the aim of causing a *reduction* in IF expression, rather than complete silencing.

Silencing observed at the mRNA level was reflected at the protein level. Strong reductions in GFAP and Vim expression were evident in astrocytes under all resting and activation conditions. Substantial Lcn2 knockdown was also observed. Thus, low doses of siRNA-3WJs are capable of downregulating astrogliosis-associated molecular markers at both mRNA and protein levels in astrocytes. However, the broader ramifications of such an intervention require further study.

Toward this end, we also examined the effect of 3WJ treatments on the expression of several pro-inflammatory cytokines and markers. *Il6* and *Nos2* expression levels, each found to be elevated in LPS+IFN-γ-treated low-FBS astrocytes, were unaffected by the substantial reductions in *Gfap*, *Vim*, or *Lcn2* afforded by our siRNA-3WJs. This may be because of the noise inherent in qPCR quantification of these low-abundance (high-threshold cycle [Ct]) transcripts masking change, or incongruities between the kinetics of protein knockdown versus subsequent secondary effects on cytokine/chemokine mRNA expression, and the time point at which these are assayed. However, an effect on the expression of these signaling mediators is not necessarily a certainty following the knockdown of our selected targets, particularly the IFs. IL-6 and nitric oxide (NO) synthesis play roles upstream of the induction of IFs,^81^, ^100^ so downregulation of GFAP/Vim is likely to be too late an intervention in the astrogliosis response to modulate the pro-inflammatory polarization of astrocytes. Lcn2, however, is known to be a secreted autocrine/paracrine mediator of astrogliosis, induced via activation of signaling pathways such as STAT3 or NF-κB, but also able to activate those same pathways upon secretion and subsequent binding to the 24p3R receptor on astrocytes.^13^, ^59^, ^101^ Despite this causal relationship between Lcn2 induction and astrocyte reactivity,^64^ our studies revealed no obvious consequence to Lcn2 ablation with respect to changes in cytokine (or *Nos2*) or IF expression, by qRT-PCR, western blot, immunofluorescence, or ELISA, in direct knockdown experiments. Again, this might be a question of induction/knockdown kinetics with induction of reactivity via stimulation with a large LPS+IFNγ dose potentially being sufficiently overwhelming to mask or inhibit therapeutic actions resulting from siRNA-3WJ treatment.

As a means to further investigate this hypothesis, we sought to investigate the paracrine mediation of reactivity in resting astrocytes by reactive astrocytes, ostensibly via secreted Lcn2 (among other actors). This was accomplished by transferring media conditioned by activated astrocytes to non-reactive astrocytes and characterizing the extent of the induced secondary reactivity. ACM obtained from LPS+IFN-γ-stimulated astrocytes (aACM) was found to induce a significant induction in *Lcn2* and *Nos2* expression in recipient resting astrocytes. These genes are significant in Lcn2-mediated reactivity because Lcn2 is known to upregulate NO production, which in turn leads to astrogliosis-associated GFAP overexpression.^59^, ^81^ Moreover, this apparent induced reactivity was completely inhibited by treating the conditioning (stimulated) cells with L12-3WJ, with the mRNA levels of *Lcn2* and *Nos2* in the aACM-treated astrocytes found to be reduced to controls. ELISA measurements performed on the conditioned media of reactive astrocytes confirmed that they do significantly upregulate the secretion of pro-inflammatory Lcn2 upon activation, and that this secretion is ameliorated by treatment with L12-3WJ. The levels of secreted Lcn2 correlate well with the subsequent induction of *Lcn2* and *Nos2* expression in recipient astrocytes. These data support the hypothesis that Lcn2, as secreted by reactive astrocytes, can act as a significant contributor to the propagation of inflammation and astrogliosis, and that a therapeutic intervention employing anti-*Lcn2* 3WJs can ameliorate this response. Nevertheless, other secreted factors are likely to play unique or synergistic roles in this phenomenon. It is noteworthy that ACM from both mock- and N03-treated astrocytes can decrease the *Lcn2* mRNA induction in recipient cells despite little effect on Lcn2 secretion from the donor cells. Clearly other mechanisms of activation/deactivation remain to be elucidated, and optimization of the timing of the ACM transfer is required to better observe the activating effects of transferred secreted Lcn2.

To investigate the efficacy of L12-3WJ *in vivo*, we employed an experimental model of moderate contusive SCI. Local delivery of a relatively low dose (10 μg) of nuclease-resistant 2′F-L12-3WJ to the injury immediately after injury significantly reduced Lcn2 expression compared with control mice. This downregulation was evident at both mRNA and protein levels. Notably, the use of a comparable dose of 2′F-N03-3WJ as a negative control did not induce any significant non-specific effects in Lcn2 expression, nor did lipofectamine injection only.

In summary, our data prove that the basic siRNA-3WJ construct is a potent, non-toxic, and non-immunogenic platform by which to deliver RNAi-based therapeutics in the CNS niche. Specifically, we have demonstrated the efficacious knockdown of several astrogliosis-associated genes, with significant reductions in mRNA and protein expression using relatively small doses of siRNA-3WJ. Moreover, knockdown was highly target-specific with obvious off-target silencing negligible compared with equivalent doses of commercial siRNA pools. Knockdown of Lcn2 resulted in a concomitant decrease in the secretion of this neuroinflammatogen, thus impeding the ability of reactive astrocytes to spread inflammation and astrogliosis. The silencing efficacy of the platform was mirrored in preliminary *in vivo* experiments, with a significant mitigation of the early Lcn2 peak observed in contusion SCI. Although these preliminary studies utilized very basic siRNA-3WJ constructs, the multifunctional and modular nature of the platform lends itself to future optimization for more specific *in vivo* applications in the CNS by incorporating further therapeutic or targeting moieties.

## Materials and Methods

### Materials

Oligonucleotides (desalted) were obtained from either Invitrogen or Sigma, whereas siRNAs (mouse anti-*Gfap*, anti-*Vim*, and anti-*Lcn2* ON-TARGETplus SMARTpools, and the ON-TARGETplus Non-Targeting pool) were obtained from Thermo Scientific. All RNA solutions and buffers were prepared in RNase-free water.

### Cell Culture

NSCs to be differentiated into astrocytes were isolated from the subventricular zone of adult C57BL/6 mice as previously described.^102^ NSCs were cultured as neurospheres in NeuroCult basal medium (mouse; STEMCELL Technologies) containing 10% NeuroCult proliferation supplement (mouse; STEMCELL Technologies), 2 μg/mL heparin (STEMCELL Technologies), 20 ng/mL recombinant human EGF (PeproTech), 10 ng/mL recombinant human FGF-basic (FGF2; PeproTech), and 100 U/mL penicillin-streptomycin (GIBCO), and passaged every 3–5 days.^103^ High-FBS/FGF2-free astrocyte differentiation was performed in media prepared using low-glucose DMEM (1 g/L glucose; GIBCO) with 10% FBS (GIBCO), 1 mM glutamine, and 100 U/mL penicillin-streptomycin. Low-FBS/+FGF2-cultured astrocytes were grown in high-FBS medium supplemented with 50 ng/mL FGF2 for the first 7 days before switching to a low-FBS version (1% FBS) supplemented with N-2 Supplement (1× final concentration; Life Technologies) and 50 ng/mL FGF2. “Activation” versions of each media were also prepared, each containing 2 μg/mL LPS (Sigma-Aldrich) and 3 ng/mL murine IFN-γ (PeproTech). All proliferating cell lines were routinely checked for *Mycoplasma* contamination. NSC neurospheres were enzymatically dissociated into single cells using Accumax (Sigma-Aldrich) and plated on poly-D-lysine (PDL)-coated plastic plates or glass coverslips at 80,000 cells/cm^2^ prior to differentiation in astrocyte media, yielding approximately 70%–90% astrocyte confluency at the later time of analysis (FGF2-free media yielding lower confluences). All cells were cultured at 37°C in a humidified atmosphere of 5% CO_2_.

### Immunofluorescence and Confocal Microscopy

Cells grown on PDL-coated glass coverslips were fixed in 4% paraformaldehyde for 10 min at room temperature at the appropriate time point post-differentiation or post-transfection, following 1 hr of serum deprivation. The coverslips were washed three times with 1× PBS before being incubated in blocking solution (1× PBS containing 0.1% Triton X-100 and 5% normal goat serum [NGS; Sigma]) for 60 min at room temperature. Fixed cells were subsequently incubated in the same blocking solution containing the desired primary antibodies (Table S2) overnight at 4°C. The following day, the cells were washed in PBS and incubated in blocking solution containing the appropriate fluorochrome-conjugated secondary antibodies (1:1,000 dilution) for 60 minutes at room temperature. Coverslips were subsequently washed again with PBS before cell nuclei were counterstained with a 1 μg/mL solution of DAPI (Roche) in PBS. After a final wash, coverslips were mounted onto slides with fluorescent mounting medium (Dako). Immunofluorescence images were acquired at ×20 and ×40 magnification using a Leica DM6000 epifluorescent microscope, whereas confocal images were acquired on a Leica TCS SP5 Microscope. Images were processed using the Fiji software package,^104^ and three-dimensional (3D) reconstructions generated using Volocity (PerkinElmer).

### ELISA

Levels of secreted murine IL-6, TNF-α, and Lcn2 were quantified using sandwich ELISA development kits (IL-6 and TNF-α [PeproTech], Lcn2 [R&D Systems]). 2 × 10^6^ NSCs were seeded in 10 mL of astrocyte medium on PDL-coated T25 plates. Medium was refreshed every 3 days, for which 3 mL was used for the assay. At 15 DIV, cells were exposed to the activation medium. Before activation, medium was collected (i.e., 0 hr activation). Medium was collected at 6, 12, 24, and 48 hr post-activation. Collected medium was spun for 15 minutes at 2,000 × *g* at 4°C, and supernatant was collected and stored at −80°C. To quantify IL-6, TNF-α, and Lcn2, we used 96-well microplates (Nunc-Thermo Scientific) to run the experiments. Eight-point standard curves were generated by using serial dilutions of standard solutions of each protein, diluted with either the diluent or the astrocyte medium used to differentiate NSCs into astrocytes. This has been performed to check whether the medium can interfere in the detection of the cytokines of interest. Data analysis was performed by interpolating unknown concentrations with the recombinant mouse IL-6, TNF-α, or Lcn2 standard curves after nonlinear regression fit, using the sigmoidal dose-response variable slope curve on log-transformed data (four-parameter logistic regression).

### qRT-PCR

Total RNA was extracted from cells using TRI Reagent (Sigma) and the protocol provided by the supplier. RNA was quantified using a NanoDrop 2000 spectrophotometer (Thermo), with purity assessed by means of A_260_/A_280_ and A_260_/A_230_ ratios. From 500 ng of total RNA, cDNA was generated using a High-Capacity cDNA Reverse Transcription kit (Life Technologies) using random hexamer primers. qRT-PCR was subsequently performed using TaqMan reagents (TaqMan Fast Universal PCR Master Mix [2×] and FAM-labeled TaqMan Gene Expression Assays) (Table S3) and an Applied Biosystems 7500 Fast real-time PCR system. Where possible, exon-spanning TaqMan probes were used to control for possible genomic DNA contamination. Unless otherwise noted, mouse *Gapdh* (VIC-labeled; Life Technologies) was used as a housekeeping reference gene for determining relative gene expression using the 2^−ΔΔCt^ method.^105^ Each biological sample was measured in triplicate.

### Western Blots

Cells were collected from six-well plates at appropriate time points post-transfection (refer to text). Proteins were extracted from the pellets by solubilization in radioimmunoprecipitation assay (RIPA) buffer (Abcam) with the addition of Complete Protease Inhibitor Cocktail (Roche) and Halt Phosphatase Inhibitor Cocktail (Thermo Scientific), as well as 1 mM phenylmethanesulfonyl fluoride (Sigma), to inhibit serine proteases. Protein quantification was performed using a Pierce BCA Protein Assay kit (Thermo Scientific).

Sample volumes equivalent to 20 μg of total protein were mixed with NuPAGE LDS Sample Buffer (Life Technologies), NuPAGE Sample Reducing Agent (Life Technologies), and distilled water prior to being heated at 95°C for 5 min. Proteins were separated by SDS-PAGE in a 10% gel using a Novex Bolt Mini Gel system and running buffer (Tris 25 mM, glycine 192 mM, SDS 10%) before being transferred onto Immobilon-P polyvinylidene fluoride membranes (0.45-μm pore size, methanol equilibrated; Millipore) in transfer buffer (Tris 25 mM, glycine 192 mM, methanol 20%) for 1 hr 45 min at 4° using a Novex Bolt Mini Blot Module. A SeeBlue Plus2 standard (Life Technologies) was used to estimate protein sizes, and transfer was confirmed by Ponceau S (Sigma) staining. For immunoblot analysis, membranes were blocked 1 hr at room temperature with 0.1% Tween 20 and 5% dried skimmed milk powder (Marvel) in PBS (pH 7.4) and then incubated with primary antibodies overnight at 4°C (Table S4) diluted in antibody solution (0.1% Tween 20, 5% dried skimmed milk powder in PBS) to the appropriate concentration. After washing with PBS/0.1% Tween 20, the membranes were incubated with the species-appropriate horseradish peroxidase-conjugated secondary antibodies (Thermo Scientific) for 1 hr at room temperature at a 1:10,000 dilution in antibody solution. Immunoreactivity was revealed using Western Lightning Plus-ECL (PerkinElmer) and imaged using a Bio-Rad ChemiDoc XRS+ system with Image Lab 5.1 software (Bio-Rad). Densitometry measurements were performed using Fiji, with each protein band being normalized to the β-actin loading controls.

### 3WJ Design and Construction

pRNA-based siRNA-3WJs were designed as per the schematic illustrated in Figure 3A, with an siRNA sequence of interest appended to one of the arms of the pRNA 3WJ core as previously established.^43^ The siRNA sequences employed in the 3WJs described herein were adapted from those used in the commercially obtained anti-*Gfap*, anti-*Vim*, anti-*Lcn2*, and non-targeting siRNA pools used as positive/negative controls in these experiments (Table S1). Strands ***A*** and ***C*** of the 3WJ were designed to contain the sense and antisense siRNA sequences, respectively.

Assembly of the siRNA-3WJs was performed according to previously reported techniques.^106^ The shorter, 20-nt ***B*** RNA strand is common to each of the 3WJ structures and so was purchased from a commercial vendor as an oligonucleotide stock. The longer, approximately 41-nt ***A*** and ***C*** RNA strands of each 3WJ were obtained as double-stranded DNA (dsDNA) templates from which the appropriate RNA strands could be prepared by *in vitro* transcription. The DNA templates were purchased as single, complementary strands, made up as stock solutions of approximately 100 μM per strand in tris/EDTA (TE) buffer, and equimolar amounts of each strand annealed together in 1× tris/magnesium/saline (TMS) buffer using a thermal cycler (5 min at 80°C before cooling by 1°C every 30 s until reaching a minimum of 4°C). Each dsDNA template was designed with a 5′ T7 RNA polymerase promoter sequence (**TAA TAC GAC TCA CTA TT**G G) to facilitate efficient transcription. Template concentrations were quantified using a NanoDrop spectrophotometer.

Transcription of the ***A*** and ***C*** RNA strands was performed using a TranscriptAid T7 High Yield Transcription Kit (Thermo Scientific) using the accompanying protocol. RNA products were isolated and purified by DNase treatment and subsequent denaturing polyacrylamide gel electrophoresis (10% polyacrylamide (w/v) gel with 8 M urea in tris/borate/EDTA [TBE] buffer). RNA bands of the appropriate length were identified by UV shadowing, cut from the gel, and diced into small pieces that were transferred to 1.5-mL Eppendorf tubes. The pieces were submerged in an elution buffer (0.5 M ammonium acetate, 0.1 mM EDTA, 0.1% SDS) and heated to 37°C water bath for 2 hr. The eluate was subsequently transferred to new tubes and stored on ice while the gel pieces were subjected to a further hour of elution using a fresh aliquot of elution buffer. All fractions of each eluate were then combined into the same tube, and the eluted RNA precipitated by the addition of 1/10 equivalent volumes of 3 M sodium acetate (pH 5.2) and 2.5 equivalent volumes of 100% ethanol. The solutions were mixed well and stored at −20°C overnight prior to centrifugation (16,500 × *g* for 30 min at 4°C), removal of the supernatant, and washing of the pellet in 500 μL of cold 75% ethanol with a further centrifugation (16,500 × *g* for 15 min at 4°C). The supernatant was again discarded and the pellet briefly dried in a vacuum concentrator prior to storage at −20°C until ready for siRNA-3WJ assembly.

Before assembly, the ***A*** and ***C*** transcribed RNA strands and the ***B*** strand oligonucleotides were re-dissolved in RNase-free water, and the concentration of each solution quantified using a NanoDrop. Where desired, RNA strands were labeled with a fluorophore (i.e., Cy3) using a Label IT Nucleic Acid Labeling Kit (Mirus BioScience) according to the supplier’s protocol with subsequent ethanol precipitation, re-dissolution, and re-quantification. Transcription and labeling success was assayed using an additional PAGE gel, running approximately 200 ng of each RNA strand in lanes alongside an Ultra Low Range dsDNA ladder (Thermo Scientific) by which to ensure each RNA strand was pure and of the correct length. Fluorescently labeled bands were identified using a ChemiDoc XRS+ system with the appropriate excitation wavelength, whereas non-labeled bands were identified by UV transillumination following staining with ethidium bromide or GelRed.

Assembly of each 3WJ was achieved by combining equimolar amounts of the appropriate strands ***A*** and ***C*** with strand ***B*** in 1× TMS buffer, typically on a 50-μL scale in a 200-μL PCR tube. The solutions were then subjected to an annealing temperature profile in a thermal cycler: heated to 80°C and held for 5 min before cooling 1°C every 30 s until reaching a minimum temperature of 4°C. Assembled 3WJs were purified of excess single RNA strands or misassembled by-products using *native* PAGE (10% polyacrylamide in TBM buffer, run at 4°C to inhibit thermal denaturation). The 3WJ bands are identified using UV shadowing cut from the gel and eluted as per the single-strand transcription products, albeit with a non-denaturing elution buffer (0.5 M ammonium acetate, 10 mM MgCl_2_). The assembled 3WJs were ethanol precipitated from the collected eluate, re-dissolved in RNase-free water, and quantified using a NanoDrop. Success of assembly was assayed by running 100 ng of each 3WJ in an additional non-denaturing polyacrylamide or agarose gel alongside the Ultra Low Range DNA Ladder, as well as samples of single ***A***, ***B***, and ***C*** strands and annealed two-strand constructs. Correctly assembled 3WJs showed lower gel mobility than did double- or single-stranded RNAs (see Figure 3B) and were diluted to a stock concentration of 5 μM in 1× TMS buffer solution for subsequent use. Further size characterization was undertaken using dynamic light scattering: 1 μM solutions of the 3WJs in 1× TMS were measured using a Zetasizer Nano ZS (Malvern Instruments).

A triple-siRNA functionalized version of the 3WJ, LGV1-3WJ, was similarly prepared from three *in vitro*-transcribed RNA strands, such that each arm possessed an siRNA moiety corresponding to the L12-, G10-, and V10-3WJs (Figure S3A). Assembly was likewise monitored electrophoretically (Figure S3B).

Whereas 3WJs intended for *in vitro* use were constructed from unmodified RNA, those made for *in vivo* use were assembled from RNA strands incorporating 2′-fluoro modified pyrimidines to afford greater nuclease resistance. Appropriately modified ***A***, ***B***, and ***C*** strands were prepared from DNA templates via *in vitro* transcription using a mutant polymerase (Durascribe T7 transcription kit; Epicenter) and further processed as per the unmodified analogs.

### *In Vitro* Transfection of NSC-Derived Astrocytes with siRNA/siRNA-3WJs

Cellular delivery of RNAs was accomplished via transfection using the liposomal transfection agent Lipofectamine RNAiMAX (Life Technologies). Silencing experiments under resting conditions were performed on astrocytes after 15 days differentiation *in vitro* using siRNA or siRNA-3WJ concentrations of 5, 0.5, and 0.05 nM (preliminary tests using primary astrocytes indicated that an optimal trade-off between silencing and off-target effects might exist in this range; data not shown). Immediately prior to transfection the astrocyte differentiation medium was replaced with low-glucose (1 g/L) DMEM serum-free medium per well. Transfections were performed according to the protocol accompanying the Lipofectamine agent. In brief, 5 μM stock solutions of the siRNAs or siRNA-3WJ of interest were diluted in DMEM and combined with an equivalent volume of DMEM containing Lipofectamine RNAiMAX (5 μL of Lipofectamine per well of a six-well plate). The solution was mixed well and allowed to incubate for 10 min before being added to cells such that the final concentration of siRNA/siRNA-3WJ was 5, 0.5, or 0.05 nM, as desired, in a 3 mL final volume of serum-free medium. Each experiment included mock transfections (Lipofectamine only, no RNA) and untreated control wells (DMEM only, no RNA or Lipofectamine). The transfection medium in each well was replaced with FBS-containing astrocyte differentiation medium 6 hours post-transfection. RNA samples were collected 48 hours post-transfection and proteins collected at various time points post-transfection, according to previously described methods. In order to investigate the effect of the RNAs on reactive astrocytes, we repeated the experiments (at 5 nM siRNA concentrations only) under activation conditions. Astrocytes were stimulated with LPS+IFN-γ-containing media for 48 hours prior to (*therapeutic*) or concurrent with and following (*preventative*) transfection at 15 DIV, with total RNA/protein collection performed as per the unstimulated samples.

### Cytotoxicity Assay

The toxicity of siRNA-3WJ treatment was ascertained using a LDH-Cytotoxicity Assay Kit II (Abcam), following the instructions provided. Toxicity/viability was normalized to non-transfected controls (100% viability) and samples lysed with a 10% solution of Triton X-100 (0% viability). Assays were performed on the supernatant of low-FBS/+FGF2 astrocytes grown in six-well plates, at time points of 24, 48, and 120 hr post-transfection. The 48- and 120-hr time points included a medium change at 6 hr post-transfection.

### Conditioned Medium Experiments

Conditioning astrocytes, activated with or without a concomitant transfection with 5 nM L12-3WJ, were washed twice with PBS and had their medium exchanged for DMEM (low glucose, 100 U/mL penicillin-streptomycin) at 24 hr post-transfection/activation. At 48 hr post-transfection/activation this ACM was spun at 600 × *g* for 10 min to remove debris; some of this medium was collected for ELISA measurements, whereas the remainder was transferred to recipient resting astrocytes. After a further 24 hr, the RNA of these target astrocytes was collected for qPCR analysis.

### Contusion Model of SCI in Mice and siRNA-3WJ Injections

All experimental procedures were performed in accordance with the Animals (Scientific Procedures) Act 1986 Amendment Regulations 2012 following ethical review by the University of Cambridge Animal Welfare and Ethical Review Body (AWERB). Animal work was covered by the PPL 700/8840 (S.P.). A total of n = 20 adult 4- to 6-week-old male C57BL/6 mice (Charles River) were deeply anesthetized with 2% Isoflurane in oxygen (1.5 L/min), and a single-vertebra laminectomy was performed in order to expose the spinal cord at the T12 level. A moderate injury was performed using a 70 kdyne force from an Infinite Horizon Impactor (Precision Systems and Instrumentation), as previously described by Cusimano et al.^107^ Following the injury, two different quantities (1 and 10 μg) of 2′F-modified L12-3WJ were delivered via transfection using the liposomal transfection agent Lipofectamine RNAiMAX (Life Technologies) into the parenchymal lesion center using a 5-μL syringe with a short 33G needle (Hamilton), in a total injection volume of 500 nL. Control animals received equivalent volume injections of either 10 μg of the negative control 2′F-N03-3WJ or diluted Lipofectamine RNAiMAX vehicle alone, or were part of an injury-only (sham) group. *Ex vivo* analyses were subsequently performed on three to four mice of each of the five treatment groups. Buprenorphine (Temgesic) was provided pre- and post-operatively; enrofloxacin (Baytril; Bayer) was provided pre-operatively and once daily to prevent infections. Bladders were manually expressed twice daily.

### Tissue Processing, *Ex Vivo* Analysis, and Tissue Pathology

2 days after injury/injections, mice were anesthetized with an intraperitoneal injection of ketamine 10 mg/mL (Boehringer Ingelheim) and xylazine 1.17 mg/mL (Bayer), and transcardially perfused with saline (sodium chloride 0.9%) plus 0.5 M EDTA (Sigma), followed by cold 4% para-formaldehyde (PFA) in PBS (pH 7.4). The isolated spinal cords were post-fixed in the same solution for 12 hr at 4°C. Tissues were then washed with PBS and cryoprotected for at least 24 hr in 30% sucrose (Sigma) in PBS at 4°C. Finally, samples were embedded in Optimal Cutting Temperature (OCT) compound (VWR Chemicals) and frozen using dry ice. Frozen cord blocks were placed in a cryostat (CM1850; Leica), and 25-μm-thick axial sections were cut and collected onto SuperFrostPlus slides (Thermo Scientific). A total of 10.5 mm of each cord segment, centered onto the lesion site, was cut and collected onto slides. Sections were then stored at −80°C until needed.

RNA was extracted from four tissue slides. Spinal cord segments were gently detached from the coverslips using a blade and placed into a 1.5-mL tube. Total RNA was extracted by resuspending tissues in 500 μL of TRI Reagent (Sigma) and incubating at room temperature for 10 min. Proteinase K from *Tritirachium album* (Sigma) was activated at 37°C for 10 min prior to addition to the TRI/RNA solutions at a 1:100 dilution and incubated at 55°C for 2 hr, followed by 80°C for 15 min. RNA was then isolated, reverse transcribed, and subjected to qPCR as described above.

For quantification of Lcn2^+^ volumes, tissue sections were pre-treated with peroxidase 3% for 15 min and then incubated for 1 hr at room temperature in the blocking solution (PBS, 10% NGS [Sigma-Aldrich], 0.3% Triton X-100 [Sigma]). Anti-Lcn2 primary antibody (MAB1857; R&D Systems) was diluted (1:200) in a solution of PBS + 1% NGS ± 0.3% Triton X-100 and incubated at 4°C overnight. The following day, after washing the sections with PBS 1×, the appropriate biotinylated secondary antibody was diluted in a solution of PBS + 1% NGS, 0.3% Triton X-100 and incubated for 1 hr at room temperature. “A” and “B” components of a Vectastain Elite ABC kit (Vector Laboratories) were then incubated for 1 hr at room temperature, and finally the reaction was developed using 3,3′-diaminobenzidine (DAB) following the supplier’s instructions. The reaction was blocked by washing the sections with distilled water, and sections were counterstained with hematoxylin. The sections were then dehydrated with increasing % alcohol solutions, washed in xylene (Merck), and mounted with a synthetic Eukitt mounting medium (Sigma).

Lcn2^+^ areas were outlined from a total of n = 7 equally spaced axial spinal cord sections using an Olympus BX53 microscope with motorized stage and Neurolucida software (v11.07 64-bit; Microbrightfield), and descriptive 3D spinal cord reconstructions were obtained. Lesion margins were determined by using hematoxylin staining, and data were expressed as the percentage (%) of Lcn2^+^ volumes per section (±SD).

### Statistical Methods

Statistical analyses were performed using the GraphPad Prism 6 software package. One-way ANOVA followed by Dunnett’s multiple comparison test was used for comparison of three or more experimental groups (qRT-PCR data), whereas two-way ANOVA with Bonferroni post-tests was used for multi-group comparisons under multiple conditions. For comparison of two experimental groups, Student’s t test was applied with Welch’s correction. Graphical results are expressed as the mean ± SD and textual descriptions as the mean ± SEM, typically derived from the means of n = 3 technical replicates from N ≥ 3 biological replicates. Statistical analysis was conducted at 95% confidence level. A p value less than 0.05 was considered as statistically significant (*p ≤ 0.05; **p ≤ 0.01; ***p ≤ 0.001; or ****p ≤ 0.0001 relative to controls). Specifics of the statistical analyses of each experiment are detailed in the corresponding figure captions.

## Author Contributions

Conceptualization: J.A.S., F.H., P.G., and S.P.; Methodology, J.A.S., A.B., J.V., C.A.-C., and F.H.; Investigation, Validation, and Formal Analysis: J.A.S., A.B., J.V., S. Basilico, S. Bandiera, C.A.-C., L.P.-J., and D.S.; Writing – Original Editing: J.A.S.; Writing – Review & Editing: J.A.S., A.B., and S.P.; Supervision: S.P. and P.G.; Funding Acquisition: J.A.S. and S.P.

## Supplemental Information

Document S1. Figures S1–S7 and Tables S1–S4

Document S2. Article plus Supplemental Information
