# Supplementary material for: RNA Nanotherapeutics for the Amelioration of Astroglial Reactivity
Source: Mol Ther Nucleic Acids. 2017 Nov 24;10:103–21. doi: 10.1016/j.omtn.2017.11.008 (PMC5738063; doi:10.1016/j.omtn.2017.11.008)
Supplement: Document S1. Figures S1–S7 and Tables S1–S4 [file mmc1.pdf]

OMTN, Volume *10*

Supplemental Information

RNA Nanotherapeutics for the Amelioration of Astroglial Reactivity

Jayden A. Smith, Alice Braga, Jeroen Verheyen, Silvia Basilico, Sara Bandiera, Clara Alfaro-Cervello, Luca Peruzzotti-Jametti, Dan Shu, Farzin Haque, Peixuan Guo, and Stefano Pluchino

Supplemental Information

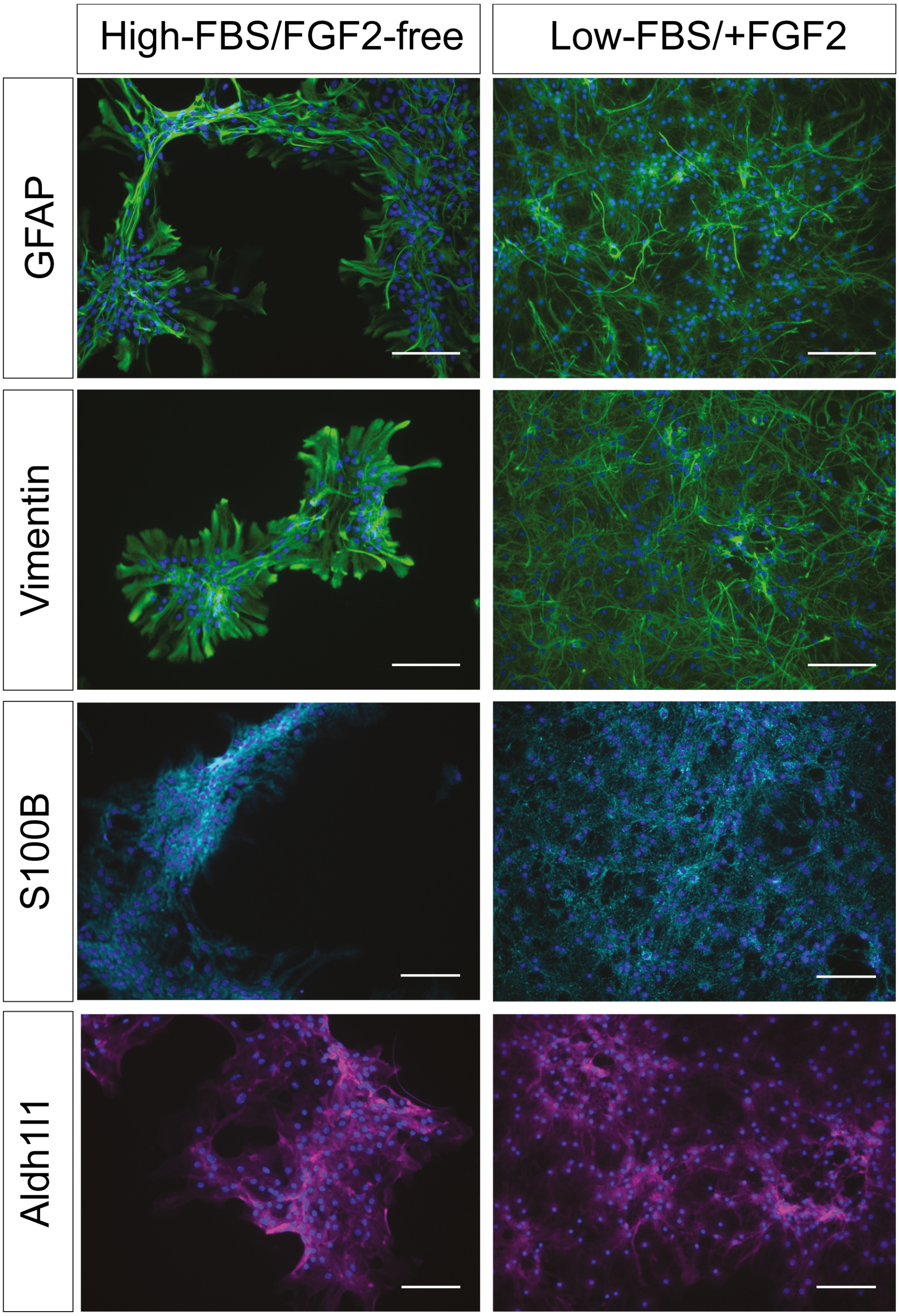

Figure S1. Immunofluorescence characterisation of high-FBS/FGF2-free versus low-FBS/+FGF2 cultured astrocytes. From top-to-bottom, comparative morphology and expression of astroglial markers GFAP, Vimentin, S100B, and Aldh1l1 in high-FBS/FGF2-free (left column) and low-FBS/+FGF2 (right column) cultured astrocytes. Nuclei counterstained with DAPI (blue). Scale bar = 100 μm.

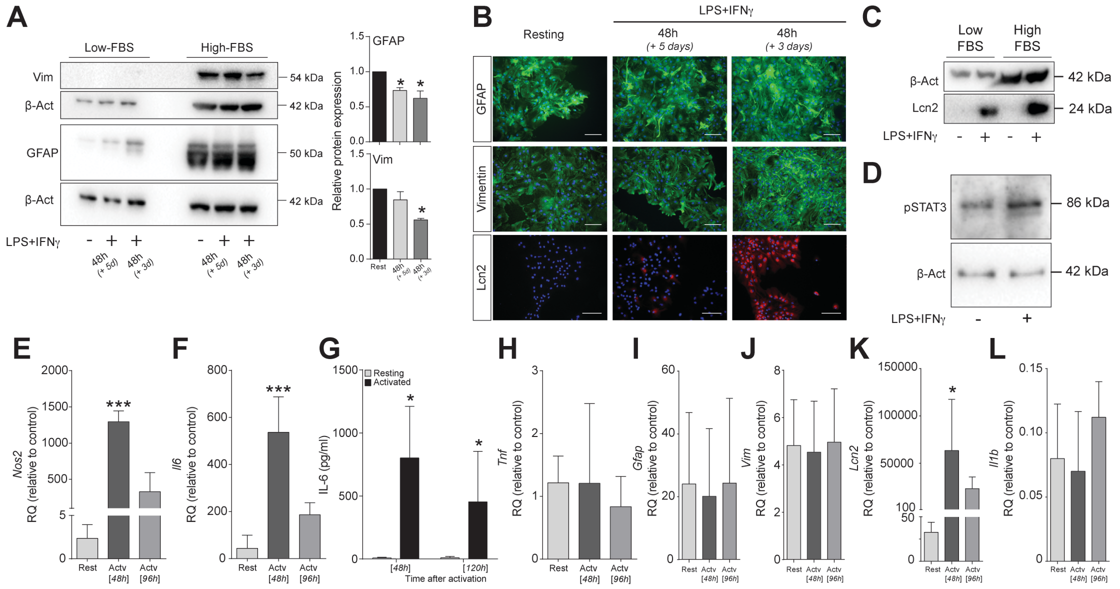

Figure S2. Response of high-FBS/FGF2-free cultured astrocytes to LPS+IFN-γ stimulation. (A) Representative Western blots showing GFAP and Vim protein expression levels 3 and 5 days after a 48h activation period, as compared to comparative conditions in low-FBS astrocytes. Densitometric quantification of GFAP and Vim Western blot expression relative to β-actin, normalised to resting conditions, is depicted alongside. **(B)** Immunofluorescence micrographs of resting and activated high-FBS/FGF2-free astrocytes showing expression of GFAP, Vim and Lcn2. Activated astrocytes imaged 3 and 5 days after a 48h activation period (GFAP/Vim: green, DAPI-stained nuclei: blue). **(C)** Representative Western blot showing expression of Lcn2 (23 kDa) upon 24h of LPS+IFN-γ exposure, in low- and high-FBS conditions. (**D**) pSTAT3 (Tyr705) in resting and activated (1h LPS+IFN-γ stimulation) high-FBS/FGF2-free astrocytes. **(E, F** and **H-K)**: qPCR quantification of **(E)** *Nos2*, **(F)** *Il6*, and **(H)** *Tnf*, **(H)** *Il6,* **(I)** *Gfap*, **(J)** *Vim*, **(K)** *Lcn2*, and **(L)** *Il1b* levels in resting and LPS+IFN-γ-treated high-FBS astrocytes (48 and 96 hours after activation). Expression relative to resting low-FBS/+FGF2 controls ($2^{-\Delta\Delta Ct}$ method), *Gapdh* reference gene. **(G)** Secreted IL-6 as measured by ELISA, resting *vs.* activated conditions, at 48h and 120h post-stimulation. All data expressed as the mean of n≥ 3 biological replicates ± SD; * p≤ 0.05, ** p≤ 0.01, *** p≤ 0.001, or **** p≤ 0.0001 relative to non-activated control samples (qPCR and Western blot: one-way ANOVA with Dunnett's multiple comparison test; ELISA: multiple t-tests with statistical significance determined using the Holm-Sidak method, α= 0.05).

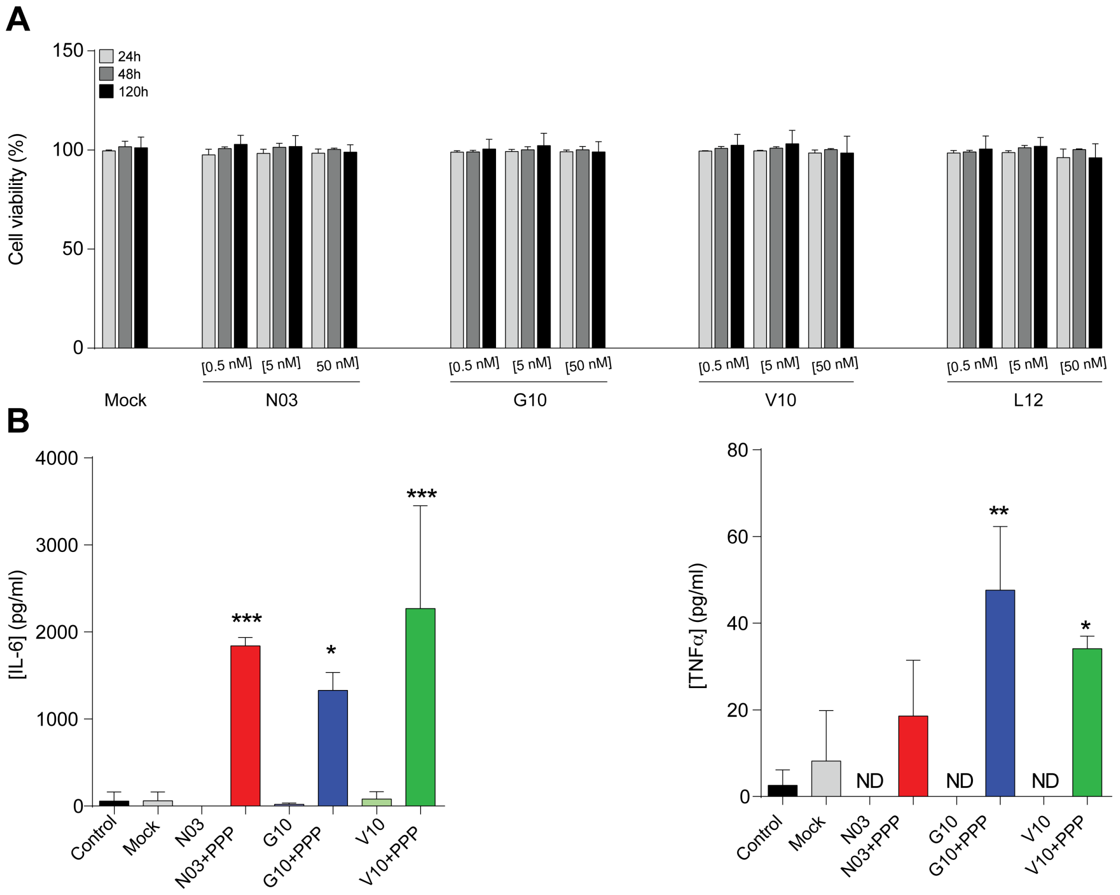

Figure S3. Dephosphorylated siRNA-3WJ nanostructures do not induce pro-inflammatory secretions and are non-toxic. (**A**) LDH cytotoxicity assay comparing the effect of 0.5, 5, and 50 nM doses of the siRNA-3WJ nanostructures employed in this study in low-FBS astrocytes. Supernatant LDH levels were determined colorimetrically at 24h, 48h and 120h after transfection, with a calibration establishing non-treated cells as 100% viable, lysed cells as 0% viable. N = 3 biological replicates, ± SD, with no statistically significant differences between treatments. (**B**) Quantification of IL-6 and TNF-α concentrations (pg/ml) in the supernatant of astrocytes treated with 3WJs with (+PPP) or without 5′-triphosphate groups. Results expressed as the mean cytokine concentration measured from n≥ 2 biological replicates ± SD; * p≤ 0.05, ** p≤ 0.01, *** p≤ 0.001, or **** p≤ 0.0001 relative to untreated control samples (one-way ANOVA with Dunnett's multiple comparison test); ND = Not Determined.

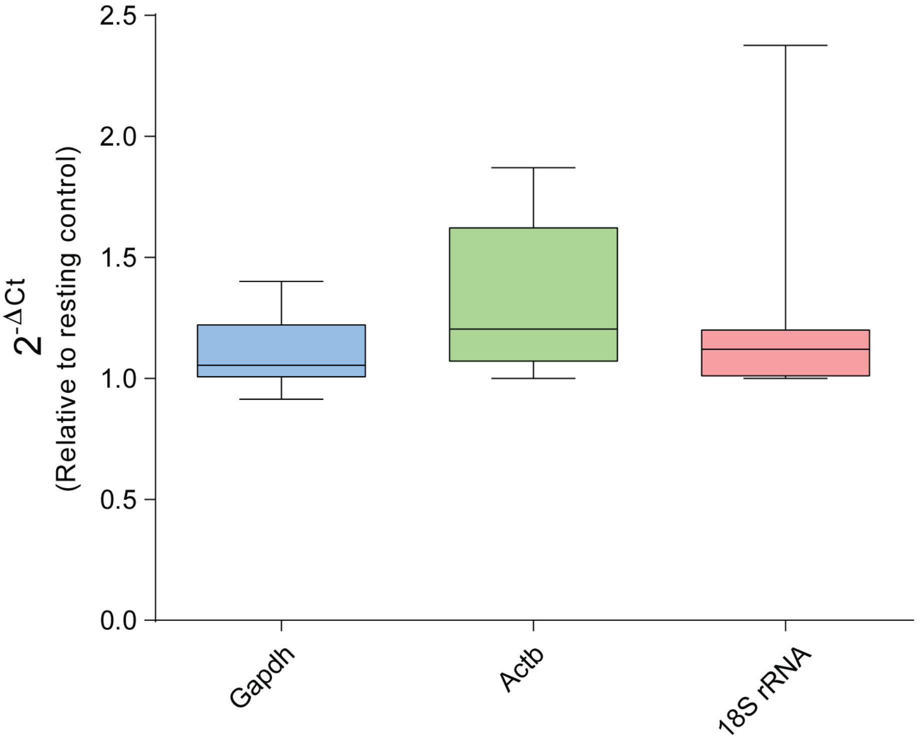

Figure S4. Reference gene expression remains stable across treatment and activation conditions. The stability of the putative genes of reference *Gapdh*, *Actb*, and *RNa18s5* were assayed across control, mock and N03-3WJ transfected treatments, under resting and both *Therapeutic* and *Preventative* activation profiles. Data are expressed as $2^{-\Delta Ct}$, where ΔCt is the mean difference in qPCR cycle threshold numbers of the reference gene between the resting control and treated sample. Results expressed as a box-and-whiskers plot of n≥ 3 biological replicates, with the box spanning the 25th to 75th percentiles and the whiskers spanning the min and max values. No statistical significance observed between reference genes.

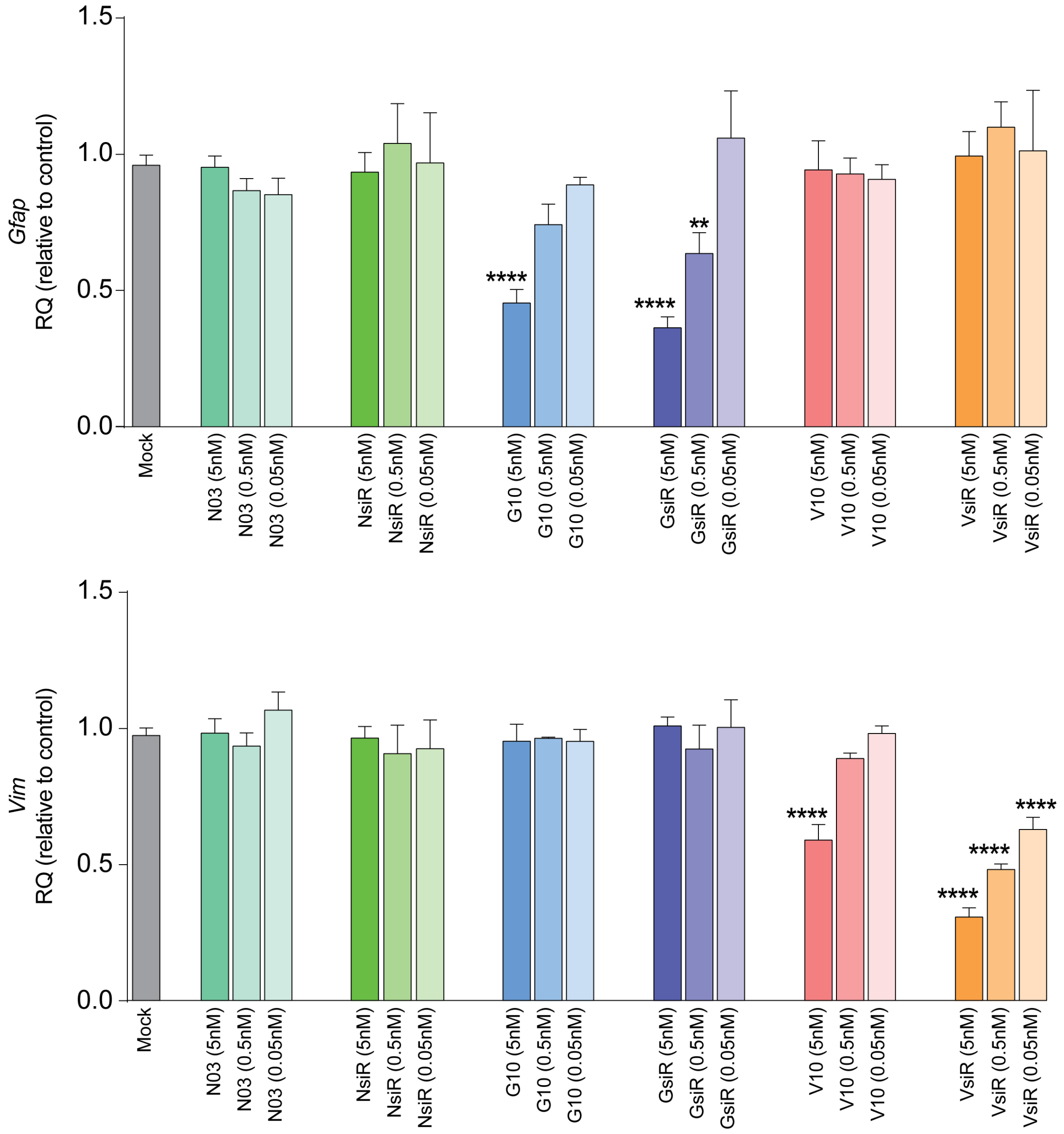

Figure S5. 3WJs knock down the expression of intermediate filament genes in a dose-responsive manner with efficiencies similar to commercial siRNA pools. (A) *Gfap* and **(B)** *Vim* mRNA knockdown measured in high-FBS/FGF-free matured astrocytes under resting conditions upon Lipofectamine RNAiMAX-mediated transfection. Non-targeted, anti-*Gfap* and anti-*Vim* 3WJs (N03, G10 and V10, respectively) are compared to analogous siRNA pools (NsiR, GsiR and VsiR, respectively). Results are expressed as the mean mRNA expression (qPCR) of n≥ 6 biological replicates ± SD; *Gapdh* reference gene; * p≤ 0.05, ** p≤ 0.01, *** p≤ 0.001, or **** p≤ 0.0001 relative to control samples (one-way ANOVA with Dunnett's multiple comparison test).

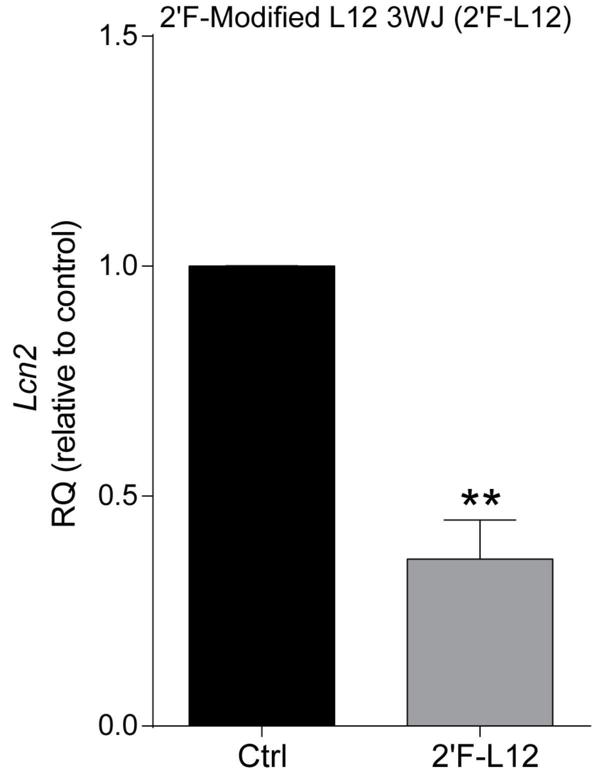

Figure S6. *In vitro* knockdown of *Lcn2* mRNA by 2'-fluoro modified L12-3WJ. qPCR data showing significant downregulation of Lcn2 mRNA by a lipofected 5 nM dose of 2'F-L12-3WJ as compared to non-treated astrocytes. Astrocytes activated with LPS+IFN-γ treatment (*Preventative* conditions), mRNA expression measure 48h after transfection. *Gapdh* reference gene. Data expressed as the mean RQ of n= 3 biological replicates ± SD; ** $p \leq 0.01$ relative to non-treated samples (unpaired t-test with Welch's correction).

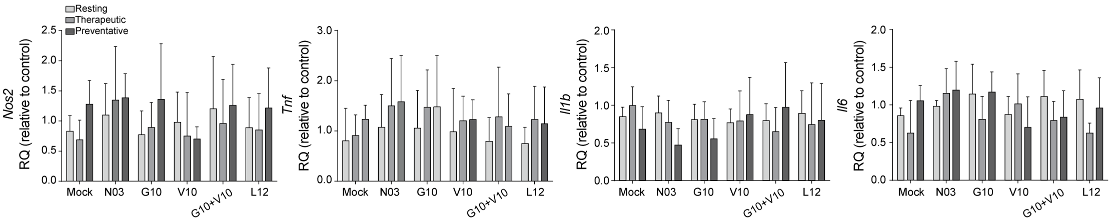

Figure S7. Neither 3WJ transfection nor knockdown of *Gfap*, *Vim* or *Lcn2* affects the expression of pro-inflammatory markers under resting or activated conditions. mRNA levels of several pro-inflammatory markers known to be upregulated in reactive low-FBS astrocytes: *Nos2*, *Tnf*, *Il1b* and *Il6*. Results are expressed as the mean mRNA expression (qPCR) of n≥ 2 biological replicates ± SD; *Gapdh* reference gene; * p≤ 0.05, ** p≤ 0.01, *** p≤ 0.001, or **** p≤ 0.0001 relative to control samples of the same activation state (two-way ANOVA with Dunnett's multiple comparison test).

Table S1. siRNA moieties of pRNA 3WJ nanostructures used in this work.

3WJ	Target	Analogous ON-TARGET siRNA[a]	Target (Sense) Sequence
G10	*Gfap* (mouse)	J-043455-10	AGC ACG AAG CUA ACG ACU A
V10	*Vim* (mouse)	J-061596-10	AGG AAG AGA UGG CUC GUC A
L12	*Lcn2* (mouse)	J-042638-12	GCG CAG AGA CCC AAU GGU U
N03	Non-Targeting	D-001810-03	UGG UUU ACA UGU UUU CUG A

[a]Individual siRNA components of the L-043455-01 anti-GFAP, L-061596-01 anti-Vim, L-042638-01 or D-001810-10 non-targeting ON-TARGET SMARTpools, as appropriate.

Table S2. Primary and secondary antibodies used in in this work.

Antibody	Supplier	Dilution
Chicken anti-GFAP	Abcam (ab4674)	1:500
Rabbit anti-GFAP	Dako (Z0334)	1:500
Chicken anti-Vimentin	Abcam (ab73159)	1:500
Rat anti-Lipocalin2	R&D Systems (MAB1857)	1:250
Mouse anti-S100B	BD Bioscience (BD612376)	1:500
Rabbit anti-Aldh1l1	Abcam (AB87117)	1:250
Rabbit anti-pSTAT3	Cell Signaling (9145)	1:100
AlexaFluor488 anti-chicken	Invitrogen	1:1,000
AlexaFluor488 anti-rabbit	Invitrogen	1:1,000
AlexaFluor546 anti-mouse	Invitrogen	1:1,000
AlexaFluor546 anti-rabbit	Invitrogen	1:1,000
AlexaFluor647 anti-rabbit	Invitrogen	1:1,000

Table S3. TaqMan Gene Expression Assay reagents used in this work.

Target Gene	TaqMan Assay
Gfap	Mm01253033_m1
Vim	Mm01333430_m1
Lcn2	Mm01324470_m1
Nes	Mm00450205_m1
Il6	Mm00446190_m1
Tnf	Mm00443258_m1
Ifnb1	Mm00439552_s1
Nos2	Mm00440502_m1
Ciita	Mm00482914_m1
Il1b	Mm00434228_m1
Tlr4	Mm00445273_m1
Ifngr1	Mm00599890_m1
Ifngr2	Mm00492626_m1
Gapd	4352339E
Actb	4319413E
18s	4352341E

Table S4. Primary antibodies used in this work.

Antibody	Supplier	Dilution
Rabbit anti-GFAP	Dako (Z0334)	1:10,000
Mouse anti-Vimentin	Abcam (ab8978)	1:500
Rat anti-Lipocalin 2	R&D Systems (MAB1857)	1:1,000
Rabbit anti-pSTAT3 (Tyr705)	Cell Signaling (9145)	1:1,000
Mouse anti-β-actin	Sigma (A1978)	1:10,000
